# Transformation of a Silicate Material for Carbon Negative Magnesia‐Based Cement via Electrochemistry

**DOI:** 10.1002/advs.202504141

**Published:** 2025-08-20

**Authors:** Anthony R. Ramuglia, Julius Scheel, Thomas Köberle, Kelly Henze, Silvia Paasch, Lairana Lima Duarte, Stefan Kaskel, Eike Brunner, Viktor Mechtcherine, Thomas Matschei, Inez. M. Weidinger, Marco Liebscher

**Affiliations:** ^1^ Institute of Construction Materials Technische Universität Dresden Georg‐Schumann‐Straße 7 01187 Dresden Germany; ^2^ Chair of Physical Chemistry Faculty of Chemistry and Food Chemistry Technische Universität Dresden Zellescher Weg 19 01069 Dresden Germany; ^3^ Chair of Bioanalytical Chemistry Faculty of Chemistry and Food Chemistry Technische Universität Dresden Bergstraße 66 01069 Dresden Germany; ^4^ Boysen‐TU Dresden‐Research Training Group TUD Dresden University of Technology Chemnitzer Str. 48B 01187 Dresden Germany; ^5^ Chair of Inorganic Chemistry I Faculty of Chemistry and Food Chemistry Technische Universität Dresden Bergstraße 66 01069 Dresden Germany; ^6^ Institute of Building materials research and Chair of Building Materials RWTH Aachen University Schinkelstrasse 3 52062 Aachen Germany; ^7^ Chair Electrochemistry Faculty of Chemistry and Food Chemistry Technische Universität Dresden Zellescher Weg 19 01069 Dresden Germany

**Keywords:** carbon‐negative, cement chemistry, electrochemistry, electrolysis, green cement, magnesia‐based cement

## Abstract

Magnesium silicate hydrate cement (M‐S‐H) can be formed from magnesium oxide (MgO) and silica phases which offer unique properties compared to traditional calcium‐based Portland cement (PC). The present study explores the transformation of a magnesium trisilicate material (Mg_2_Si_3_O_8_) into the precursor phases of M‐S‐H cement via the electrolysis of water. The investigation examines the change in pH as a consequence of water electrolysis, resulting in the dissolution of the Mg_2_Si_3_O_8_ and formation of Mg(OH)_2_ and SiO_2_. The material phases collected after dissolution are characterized via SEM, EDX, XRD, IR, ^29^Si NMR and BET analysis. The results indicate brucite accumulates in large platelet‐like structures and analysis of the residual silicate phase present after electrolysis‐induced dissolution reveal protons have replaced the Mg^2+^ ions. Amorphous SiO_2_ can be recovered from the system through pH adjustment, producing SiO_2_ with a high surface area ideal for cement production. As this process is conducted electrochemically, this approach to silicate material transformation represents an avenue toward cement manufacturing devoid of CO_2_ emissions. Through carbon‐curing, the M‐S‐H cement can constitute a carbon‐negative system. Mg_2_Si_3_O_8_, a synthetic material, serves as a model for extrapolating this processes to earth‐abundant silicate minerals enabling their potential use in large‐scale sustainable cement manufacturing.

## Graphical abstract

The transformation of a magnesium trisilicate material via water electrolysis into precursor phases necessary for magnesium‐based M‐S‐H cement is explored. The dissolution of the material mediated electrochemically is monitored, and the analysis of the resulting phases indicate their suitability as effective precursors for M‐S‐H cement. The investigation culminates into a sustainable and environmentally benign avenue toward magnesium‐based carbon‐negative cement production.

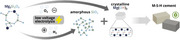

## 1 Introduction

Cement, the primary adhesive component which constitutes concrete, is the most ubiquitously produced material in the world.^[^ ^1^ ^]^ Worldwide cement manufacturing accounts for ≈8% of global CO_2_ emissions and its production is only expected to increase, exacerbating climate instability.^[^ ^2^ ^]^ Generally speaking cement is conventionally manufactured first through mixing carbonate‐containing minerals, predominately limestone (CaCO_3_) with silicates and silica (SiO_2_), or other pozzolan material (siliceous and/or aluminous compounds) and heated in a precalciner at high temperatures (850‐950 °C). The calcination of limestone results in the decomposition of CaCO_3_, releasing CO_2_ and the formation of calcium oxide (CaO). This mixture of CaO and aluminosilicates is then sintered in a rotary kiln at temperatures up to ≈1450 °C to produce composite silicates such as alite (Ca_3_SiO_5_) and belite (Ca_2_SiO_4_), with alite compromising the major active phase ≈(60–70%) of typical Portland cement (PC).^[^ ^3^ ^]^ In both cases the heat required for calcination and sintering is generated through the combustion of fossil fuels, releasing further CO_2_ among other environmental pollutants.^[^ ^4^ ^]^ The necessity to attenuate the CO_2_ produced as a byproduct of cement manufacturing has manifested in the blossoming of low carbon manufacturing techniques in the form of electrochemical cement clinker production.^[^ ^5^ ^]^ Seminal work by Ellis and Chiang^[^ ^6^ ^]^ and subsequent investigations by Berlinguette^[^ ^7^ ^]^ and others^[^ ^8^ ^]^ display the utility of employing electrochemistry in the fabrication of cement clinker precursor, in this case calcium hydroxide (Ca(OH)_2_) referred to as slacked lime, primarily from CaCO_3_. The electrochemical fabrication method results in CO_2_ abatement via the circumvention of fossil fuel ignition in the calcination step and allows the formation of a pure CO_2_ stream from the decomposed CaCO_3_ which can be effectively captured and upcycled. The transformation of CaCO_3_ into Ca(OH)_2_ is achieved electrochemically through the catalytic electrolysis of water, wherein water is oxidized to protons (H^+^), electrons (e^−^) and oxygen gas (O_2_) at the anode and reduced into hydroxide ions (OH^−^) and hydrogen gas (H_2_) at the cathode (Equations 1 and 2).

(1) $$\mathrm{Anode}:2H_{2}O\to O_{2}+4H^{+}+4e^{-}$$

(2) $$\mathrm{Cathode}:4H_{2}O+4e^{-}\to 2H_{2}+4OH^{-}$$

Recently, magnesium‐based cements and the role of magnesium oxide (MgO) in cementitious materials have garnered significant attention in the cement community. Although not a direct replacement for CaO‐based cements,^[^ ^9^ ^]^ Magnesium‐Silica‐Hydrate cements referred to as M‐S‐H, (as an aside the authors note this nomenclature is prevalent in the cement literature however it does not align with conventional chemical nomenclature) form at lower pH (10.5 vs ≈13 for PC),^[^ ^10^ ^]^ lower temperatures than typical cement^[^ ^11^ ^]^ and can be used as an alternative binder system with specific mechanical properties compared to that of CaO‐based cements.^[^ ^9^, ^10^, ^12^ ^]^ M‐S‐H is formed via calcination Mg(OH)_2_ into MgO at ≈500 °C, followed by ambient temperature mixing with amorphous SiO_2_ and water.^[^ ^13^ ^]^ Within the magnesium silicate cementitious phases, hydration of the calcinated MgO regenerates quantities of Mg(OH)_2_ within the cement. The regenerated Mg(OH)_2_ displays low energy barriers for CO_2_ absorption, resulting in a range of hydrated magnesium carbonate mineral phases, acting as a CO_2_ sink through carbon dioxide mineralization.^[^ ^14^ ^]^ This carbon‐curing produces a dense network of well‐connected carbonate microstructures throughout the silicate phases, improving the compressive strength of the M‐S‐H cement (typically ≈46.8 MPa after 28 days).^[^ ^15^ ^]^ M‐S‐H cement itself holds promise as potential matrixes in radio nucleotide retention for nuclear and heavy metal waste storage^[^ ^16^ ^]^ and its lower pH allows for the incorporation of natural fibers to supplant steel‐reinforcement.^[^ ^12b^ ^]^ However, the equivalent carbonate mineral magnesite (MgCO_3_) is geographically limited and high quality available feedstocks are typically extracted outside of the EU and imported.^[^ ^17^ ^]^ Moreover, although common and effective starting materials in modern‐day cement manufacturing, the transformation of carbonate‐based minerals (CaCO_3_ or MgCO_3_) into cementitious precursors inherently results in the release of CO_2_ during calcination or electrolysis.^[^ ^4^, ^5^, ^14^ ^]^ Siliceous minerals or silicates on the other hand, (e.g. olivine, serpentine, basalt) are globally more abundant than carbonate minerals making up ≈90% of the earth's crust^[^ ^18^ ^]^ with magnesium‐based silicates constituting a large percentage of these minerals in the form of ultramafic rock.^[^ ^19^ ^]^ The dissolution of these magnesium‐based silicates would generate the same alkali earth‐based cementitious precursors as with carbonate minerals yet result in the production of reactive SiO_2_ rather than CO_2_, representing a more environmentally benign process for cementitious material manufacturer and avoid the energy intensive need for CO_2_ upcycling. Importantly, the amorphous SiO_2_ could constitue an as excellent source of silica for the M‐S‐H, circumventing the need for outside sources of silica in the manufacturing process.

Rau et. al have demonstrated the direct dissolution of ultramafic rock and the Ca‐containing silicate mineral wollastonite for carbon capture, however the application of such minerals in cement precursor production has not yet been investigated.^[^ ^20^ ^]^ Exceptional work in the production of Mg(OH)_2_ from the magnesium silicate mineral olivine by Scott and co‐workers has been shown to be effective after dissolution of the mineral in HCl followed by subsequent filtration.^[^ ^21^ ^]^ Scott has subsequently shown the applicability of olivine as a starting material in the production of magnesium‐based cements.^[^ ^13^ ^]^ As reported, Mg‐containing silicates can be dissolved in HCl and used to produce Mg(OH)_2_. However, conducting electrolysis to generate an acidic environment in which to dissolve such minerals circumvents the need for HCl. In turn, the formation of toxic Cl_2_ gas, a byproduct of electrolysis of MgCl_2_ formed via dissolution of the magnesium‐containing minerals in the aforementioned acid, is also mitigated.^[^ ^21^, ^22^ ^]^

Intrigued by the potential application of electrochemistry in the production of M‐S‐H cement precursors, we sought to bridge these two realms of silicate mineral transformation and electrochemical cement precursor synthesis. In the present work the decomposition of the synthetic material magnesium trisilicate, Mg_2_Si_3_O_8_, into the cementitious precursor components Mg(OH)_2_ and amorphous SiO_2_ using electrochemistry is investigated. The idealized transformation of this material into M‐S‐H cement is depicted in Equations (3)–(6). Initially, the Mg_2_Si_3_O_8_ is dissolved into the constituent components Mg^2+^, SiO_2_ and H_2_O as a consequence of interacting with the protons generated electrochemically (3). Mg(OH)_2_ is formed from dissolved Mg^2+^ ions interacting with electrochemically generated OH^−^ ions (4). Calcination of Mg(OH)_2_ results in the formation of MgO and H_2_O (5). A mixture of varying quantities of MgO, SiO_2_ and H_2_O at ambient temperature produces M‐S‐H (6).

(3) $$Mg_{2}Si_{3}O_{8}+4H^{+}\to 2Mg^{2+}+3\mathrm{Si}O_{2}+2H_{2}O$$

(4) $$2Mg^{2+}+4OH^{-}\to 2\mathrm{Mg}{\left(\mathrm{OH}\right)}_{2}$$

(5) $$2\mathrm{Mg}{\left(\mathrm{OH}\right)}_{2}\overset{500^{\circ }C}{\to }2\mathrm{MgO}+2H_{2}O$$

(6) $$\mathrm{xMgO}+\mathrm{ySi}O_{2}+zH_{2}O\to M-S-H$$

The current report focuses on the dissolution of the Mg_2_Si_3_O_8_ material into its constituent components under ambient conditions employing an electrochemical system operated galvanostatically (i.e., controlled current) conditions. The collected products of the dissolution are analyzed for their applicability and utility as precursor phases in M‐S‐H cement.

## 2 Results and Discussion

### 2.1 Electrochemical Investigations

**Figure 1:**
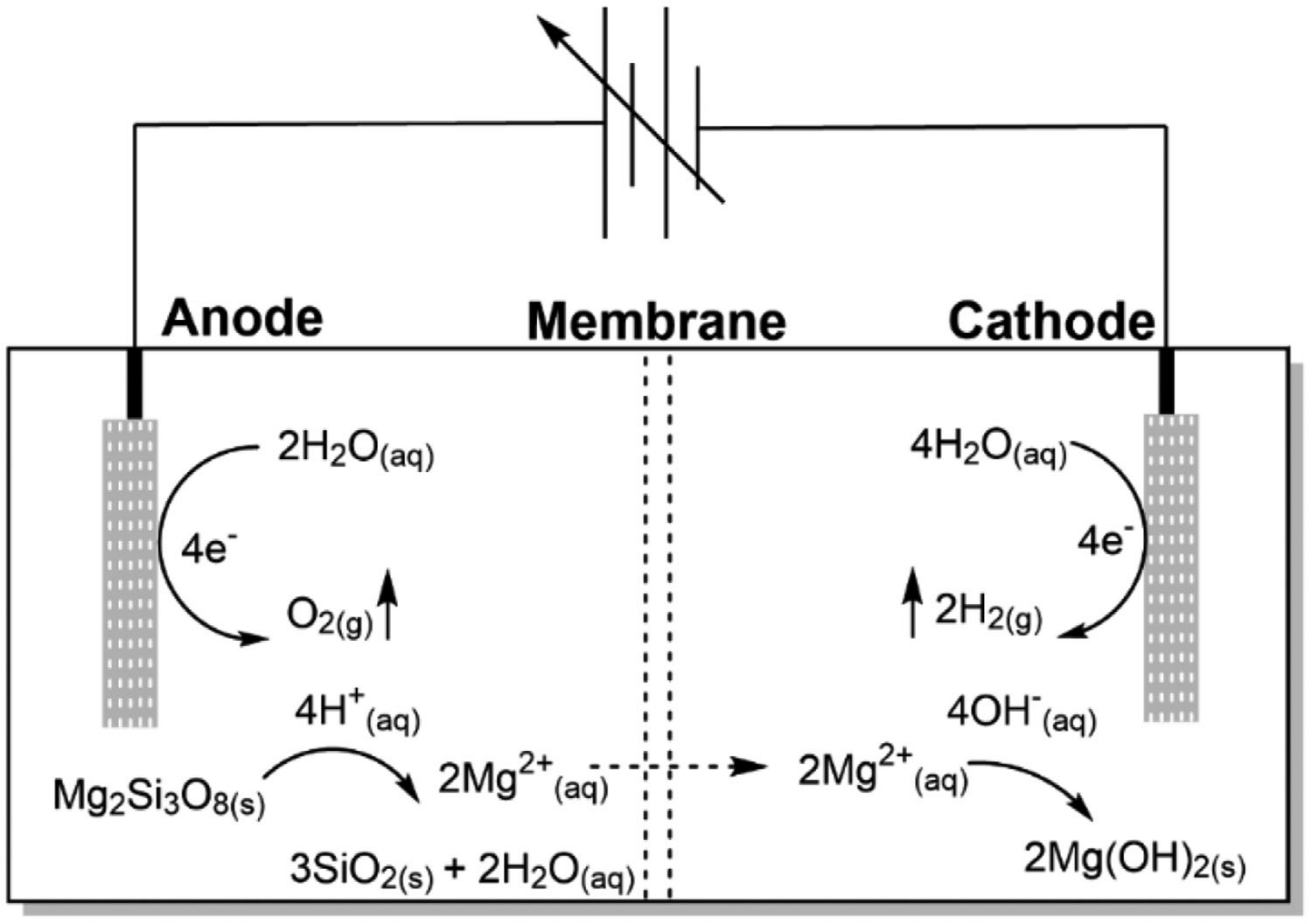
Schematic diagram of the conceptualized electrochemical “H” cell. Water is oxidized at the anode and reduced at the cathode using a DC power supply. The resulting water electrolysis products (H^+^ and OH^−^) interact with the Mg_2_Si_3_O_8_ to dissolve the silicate and produce the corresponding Mg(OH)_2_ while O_2_ and H_2_ leave as gases.

A two‐compartment electrochemical cell separated by a Nafion 115 cation exchange membrane containing 0.5 M sodium sulfate (Na_2_SO_4_) was utilized as the electrolysis system for the dissolution of the Mg_2_Si_3_O_8_ (for more details regarding the experimental setup the reader is referred to the methods section within the Supporting Information). In typical experimental measurements, Mg_2_Si_3_O_8_ was added to the anodic compartment of the electrochemical cell before galvanostatic electrolysis was initiated. A schematic diagram of the electrochemical cell and the component reactions can be observed in **Figure** **1**. Within the figure, the Mg_2_Si_3_O_8_ is dissolved in the proton‐rich environment generated by the anode to form SiO_2_, Mg^2+^ ions and water. The resulting Mg^2+^ ions diffuse through the Nafion membrane toward the negatively charged cathode where they interact with hydroxide ions to form Mg(OH)_2_. The O_2_ and H_2_ generated leave the system as gases. Importantly, the O_2_ and H_2_ gases produced as a byproduct of water electrolysis can be further utilized as fuel sources. Mixtures in the form of oxyhydrogen are well capable of reaching temperatures required for calcinating Mg(OH)_2_.^[^ ^23^ ^]^

After the addition of the Mg_2_Si_3_O_8_, the anodic compartment was continuously stirred to improve substrate dissolution. Pulse chronopotentiometry was conducted at 40 mA over 24 h, resulting in a cell voltage of ≈1.8 V vs Ag/AgCl, (Figure S1, Supporting Information). The voltage is adequate enough to overcome the thermodynamic barrier of water oxidation on Pt. In some instances, the current density is seen to dip and attenuate during electrolysis, likely due to passivation of the Nafion membrane impeding ion mobility between the two compartments (Figure S2, Supporting Information). Linear sweep voltammetry (LSV) was conducted in the presence and absence of Mg_2_Si_3_O_8_ within the system and the resulting Tafel plots indicate slightly higher overpotentials (*η*) are required to achieve the same current density for water oxidation in the presence of Mg_2_Si_3_O_8_ (Figures S3 and S4, Supporting Information, respectively). However, no early onset nor other electrochemical phenomena is observed in the presence of Mg_2_Si_3_O_8_, which suggests no additional oxidative side reactions of the substrate occur within the potential window. The dissolution of the material is therefore likely purely a result of H^+^ interaction, generated via water electrolysis. Impedance measurements in the presence and absence of Mg_2_Si_3_O_8_ at 40 mA indicate a slight decrease in solution resistance upon the addition of the substrate (Figure S4, Supporting Information) yet no change in the interfacial resistance at the anode‐electrolyte interface. The formation of inactive surface layers on the anode as a result of Mg_2_Si_3_O_8_ dissolution can therefore be excluded. The slightly higher *η* in the presence of Mg_2_Si_3_O_8_ from the Tafel plots may thus be attributed to changes in the local pH at the electrode surface, however further investigations into such changes are warranted. The electrolyzer itself effectively operates at high faradaic efficiencies (FE) of 76 %, (Figure S5 and Table S1, Supporting Information) as electrolysis solely oxidizes and reduces water. It is important to note however that although this current laboratory scale system can provide an effective proof of concept, the system itself requires further optimization, specifically in aspects of cell design. More complex open systems such as flow cells with feed and bleed liquid and gas circulation^[^ ^24^ ^]^ or systems devoid of ion exchange membranes altogether^[^ ^5^, ^25^ ^]^ circumvent issues in membrane passivation and are better suited for large scale electrolysis applications. We therefore refrain from discussing the energy metrics of the system and how they pertain to the practicality of electrolysis at scale, however the technoeconomic implications of electrolyzers for large scale cement production have been outlined elsewhere.^[^ ^6^, ^7^, ^20^ ^]^

**Figure 2:**
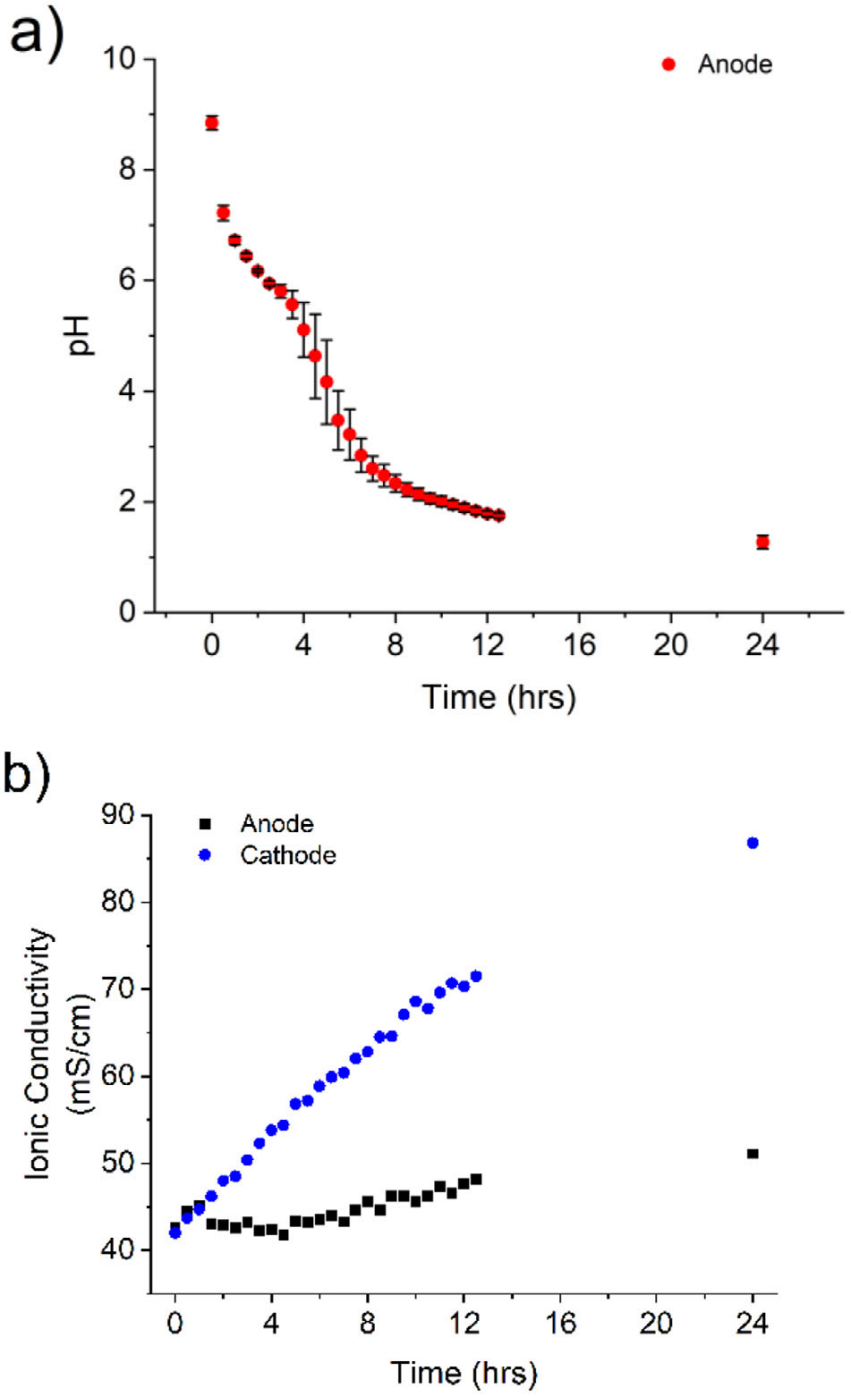
a) pH measurement taken in the anodic compartment of the electrochemical cell in the presence of Mg_2_Si_3_O_8_ during electrolysis, conducted in triplicate. Error bars represent the standard deviation between measurements. b) Ionic conductivity measurements taken in both the anodic (black) and cathodic (red) compartments during electrolysis.

The pH of the anodic compartment containing the Mg_2_Si_3_O_8_ was monitored over the course of the electrolysis and is depicted in **Figure** **2**a, with measurement replicated in triplicate (Table S2, Supporting Information). As evident from the plot, the pH is initially slightly basic (8.8), owing to the inherent basicity of the Mg_2_Si_3_O_8_. During electrolysis, the pH drops incrementally due to the oxidation of H_2_O into O_2_ and H^+^, with a measured pH of ≈3.5 after 6 h, reaching a local minimum of ≈1.8 after 12 h and tapering off at 1.1 after 24 h. Ionic conductivity measurements performed in the anodic compartment in the presence of Mg_2_Si_3_O_8_ indicate that the concentration of charged ions remains relatively stagnated, increasing only ≈10 mS cm^−1^ on the electrolysis time scale (**Figure** 2b, black trace). This trend in ionic conductivity is not observed in the absence of the starting material, wherein a larger increase in ionic conductivity is observed over the same time scale (Figure S6 and Table S3, Supporting Information). The conductivity within the cathodic compartment conversely continually increases, as OH^−^ is produced at a rate kinetically faster than the dissolution of the Mg_2_Si_3_O_8_ silicate in the anodic compartment,^[^ ^26^ ^]^ **Figure** 2b, blue trace (Table S3, Supporting Information). The difference in ionic conductivity at the anode and cathode during electrolysis can be conceptualized through the formation of H^+^ at the anode dissolving the Mg_2_Si_3_O_8_ starting material. The dissolution results in the formation of Mg^2+^ ions which are replaced with H^+^ ions in the material. The Mg^2+^ ions migrate through to the cathodic compartment, and the formation of neutral, silicic acid (Si(OH_4_)) at low enough pH, remains in the anodic compartment (vide infra). Investigations of the system at low amperages (4 mA) leads similarly to a decrease in pH albeit over much longer time scales. Higher amperages (80 mA) at similar potentials results in a more dramatic decrease of the pH on the electrolysis time scale compared to lower amperages, suggesting input current affects the speed of the electrolysis half reactions (Figure S7, Supporting Information). However, current or amperage overload errors within the potentiostat result in low‐integrity data in some instances, therefore lower amperages were required for analytical analysis from our instruments. KNO_3_ was also observed to be a viable electrolyte for this process, as galvanostatic electrolysis under identical conditions (40 mA) also results in a pH decrease over time (Figure S8, Supporting Information). Notably however, Na_2_SO_4_ more industrially practical due to its comparative cost 101 € per kg vs KNO_3_ 185 € per kg, (Sigma Aldrich).

### 2.2 Residual Silicate Phase and Product Analysis

**Figure 3:**
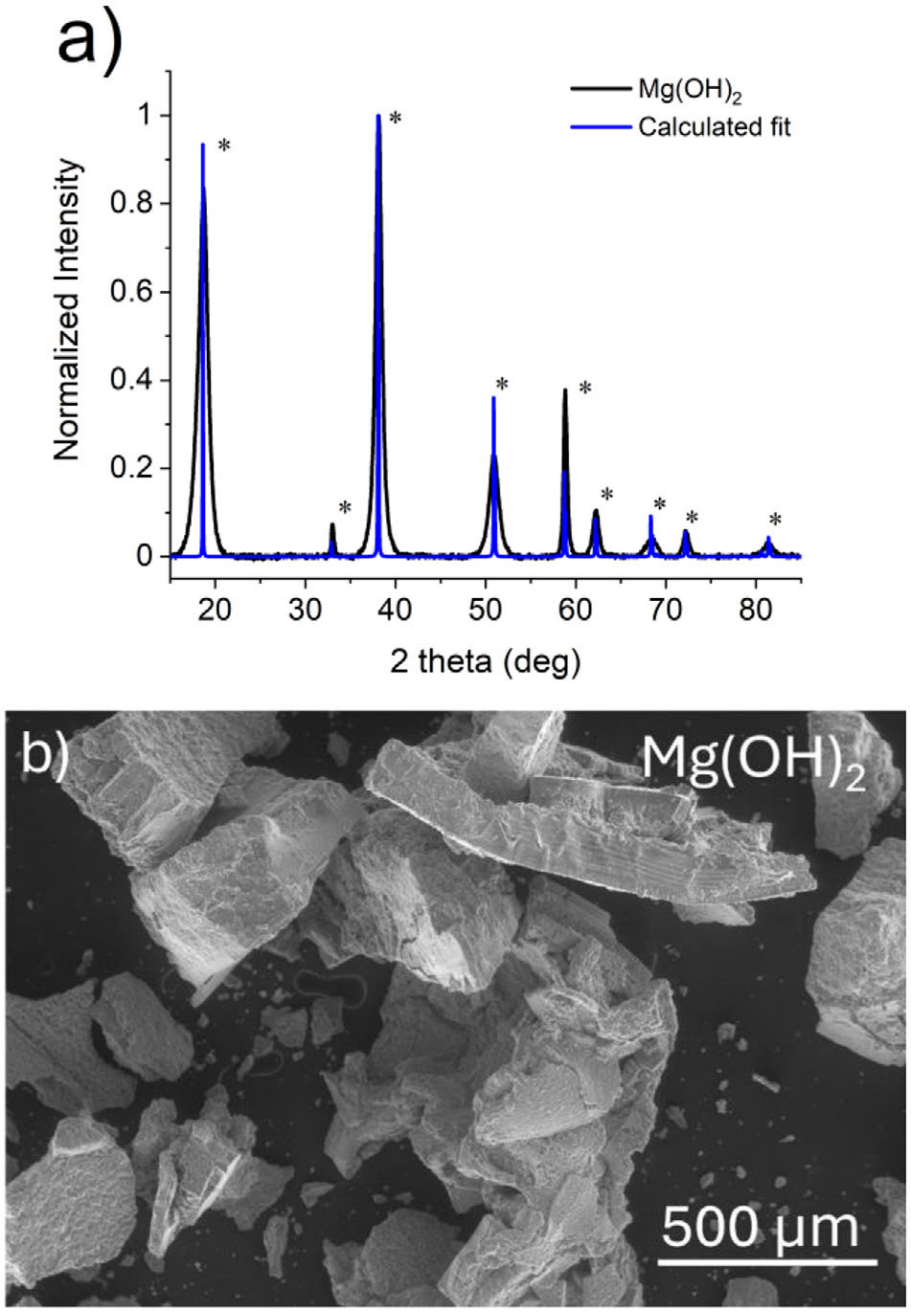
a) XRD powder diffraction of Mg(OH)_2_ formed in the cathodic compartment (black trace) and calculated Mg(OH)_2_ fit (blue trace) b) SEM imaging of the Mg(OH)_2_ formed in the cathodic compartment of the electrochemical cell after electrolysis; collected at 200 x magnification.

After chronopotentiometric electrolysis times of greater than 6 h, a white precipitate was observed at the Nafion membrane partition. With slight agitation of the cell, the white precipitate began to collect in the cathodic compartment. Electrolysis was continued to 24 h after which time the experiment was stopped, the electrolyte was removed and both the precipitate in the cathodic compartment and residual silica phase (RSP) i.e. what was initially the starting material subject to acidification in the anodic compartment, were filtered, rinsed with deionized water and let to dry overnight in the fume hood. X‐ray diffractometry (XRD) analysis confirms the existence of pure Mg(OH)_2_ (brucite) as the solid product collected in the cathodic compartment and fits excellently to the calculated data (**Figure** **3**a). Scanning electron microscopy (SEM) images of the Mg(OH)_2_ indicate large, layered platelet‐like structures spanning over 500 µm (**Figure** 3b). Fourier Transform Infrared spectroscopy (FTIR) and Raman spectroscopy also support the XRD analysis that Mg(OH)_2_ is formed in the cathodic compartment as a result of electrolysis (Figures S9 and S10, respectively).

**Figure 4:**
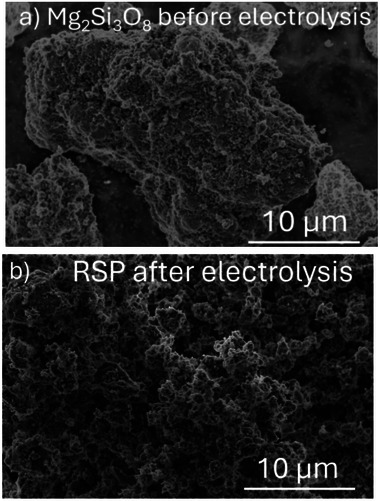
a) SEM images of Mg_2_Si_3_O_8_ starting material before electrolysis b) The residual silica phase (RSP) collected after electrolysis after 24 h; both images collected at 12000x magnification.

After 24 h of electrolysis the collected RSP was analyzed via SEM and compared to that of the Mg_2_Si_3_O_8_ starting material, shown in **Figure** **4**. The SEM images indicate the RSP is composed of clusters of small particles with void spaces in the micrometer range, indicative of the dissolution of the Mg^2+^ through acidification of the starting material.

**Figure 5:**
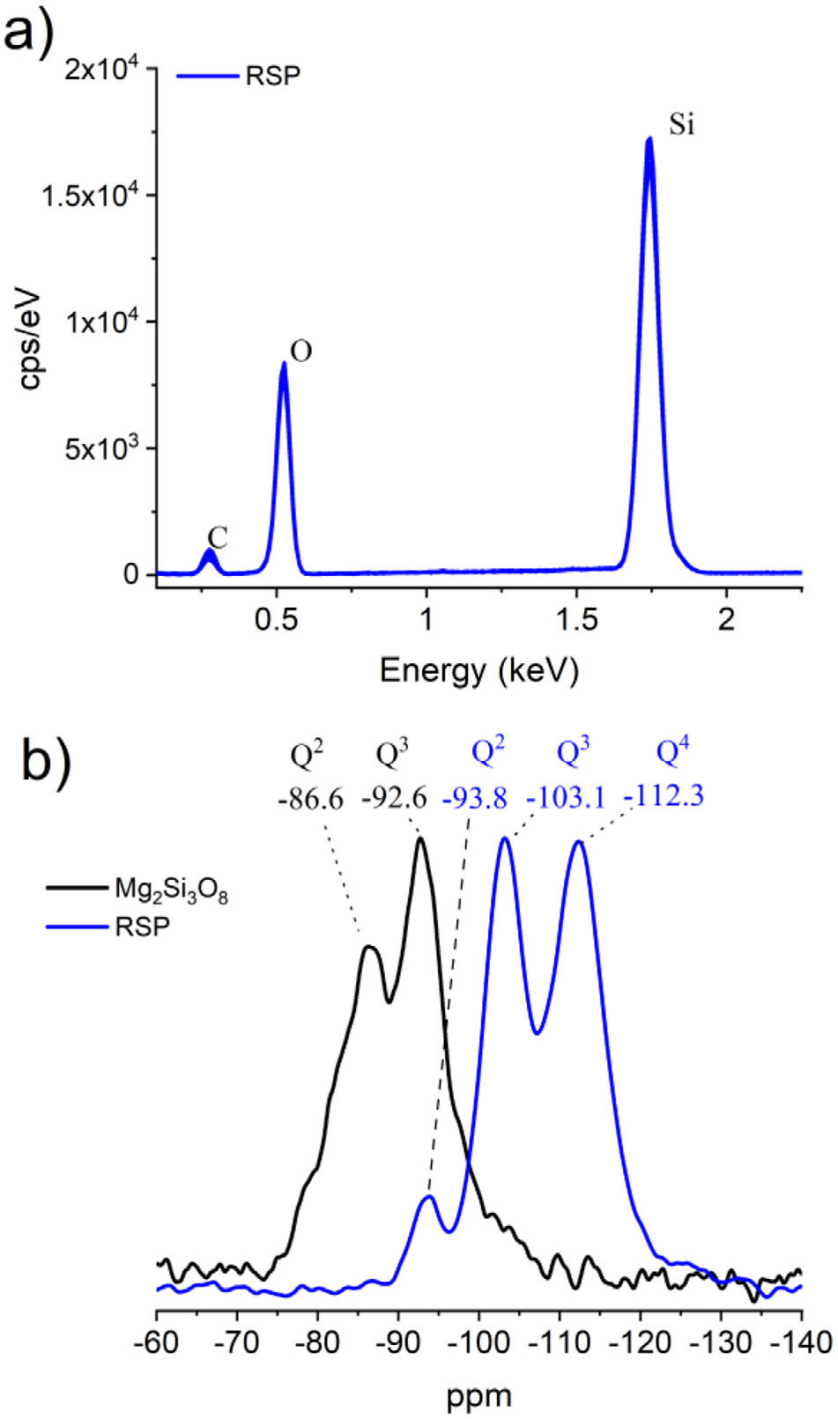
a) EDX analysis of the residual silica phase (RSP) collected in the anodic compartment of the electrochemical cell after 24 h of electrolysis b) ^29^Si MAS NMR spectra of the residual silica phase (RSP) collected in the anodic compartment of the electrochemical cell after 24 h of electrolysis, blue trace; Mg_2_Si_3_O_8_ starting material, black trace.

Energy dispersive X‐Ray (EDX) spectroscopy was utilized to elucidate the composition of the RSP collected. EDX measurements shown in **Figure** **5**a of the RSP display bands corresponding exclusively to Si and O, indicating the formation of pure silica, underlying the dissolution of Mg^2+^ ions from the starting silicate material at low pH values. The carbon band observed in the spectrum is an artifact of the signal from the EDX background (Figure S11, Supporting Information). XRD analysis of the RSP display a large, broad peak spanning from 15 to 35 ° deg on the 2 theta axis (Figure S12, Supporting Information). Magic angle spinning (MAS) ^29^Si NMR spectroscopy was performed to further analyze the structure of the Mg_2_Si_3_O_8_ starting material and its decomposed counterpart, the RSP, both depicted in **Figure** 5b (black and blue traces, respectively). ^29^Si MAS NMR can be an effective tool for understanding the structural features of silica containing materials^[^ ^27^ ^]^ and notably has been shown to be a useful method in determining the composition of silica‐based slag fly ash composites and M‐S‐H phases.^[^ ^28^ ^]29^Si NMR provides structural information regarding the coordination environment of the silicon atoms in the sample, referred to as Q*^n^*, where *n* denotes the number of bridging oxygen atoms connecting silicon atoms in the silica structure.^[^ ^29^ ^]^ For example, a Q^4^ environment corresponds to a silicon atom bonded to four oxygen atoms which in turn are connected to silicon atoms (SiO)_4_Si, forming a connected network. A Q^3^ environment corresponds to one fewer Si─O─Si linkage, typically in the form of a silanol (Si─OH), indicating (SiO)_3_Si─OH. **Figure** 5b depicts the ^29^Si MAS NMR spectra of the Mg_2_Si_3_O_8_ starting material (black trace) and RSP (blue trace). The Mg_2_Si_3_O_8_ starting material displays a signal at ‐86.6 ppm corresponding to a Q^2^ environment and a signal at ‐92.6 ppm corresponding to a Q^3^ environment.^[^ ^30^ ^]^ The observed Q^3^ signal at ‐92.6 ppm is attributed to a Si─O─Mg connection, and the Q^2^ signal at −86.6 ppm is attributed to a terminal silanol containing an Mg‐bound connection in the form of (SiO)_2_Si(OMg)(OH).^[^ ^31^ ^]^ The NMR spectrum of the RSP recovered after electrolysis displays three signals at −93.8, −103.1 and −112.3 ppm which correspond to Q^2^, Q^3^ and Q^4^ environments, respectively. Generally, an ≈10 ppm high‐field shift is observed for the formation of a Si─O─Si connection.^[^ ^32^ ^]^ In this case, the extrusion of the Mg^2+^ ions results in a shift upfield (more negative) of the Q*^n^* environments such that the Q^2^ environment of the RSP is observed at more negative ppm (−93.8 ppm) than the Q^3^ environment of the Mg_2_Si_3_O_8_ (−92.6 ppm). This signal at −93.8 ppm corresponding to the Q^2^ band of the RSP is assigned to hydroxylated geminal silanol groups (Si─(OH)_2_) in the form of (SiO)_2_Si(OH)_2_, while the signal at ‐103.1 ppm points toward a single proximal silanol environment (Si─OH) in the form of (SiO)_3_Si(OH).^[^ ^29^ ^]^ These partially hydroxylated silica environments of the RSP allude to a partial dissolution of the silicate structure and a substitution of H^+^ for previous Mg^2+^ ions after Mg^2+^ dissolution. The RSP signal at −112.3 ppm is however indicative of a more highly‐ordered Q^4^ environment, similar to those found in pure silica environments (SiO_4_)^[^ ^32b^ ^]^ corresponding to a higher degree of silica polymerization formed after the removal of the Mg^2+^ ions.^[^ ^28c^ ^]^ This suggests some amount of condensation of residual silica fragments after Mg^2+^ dissolution, observed similarly in silicate minerals subjected to acid weathering.^[^ ^33^ ^]^ Collectively, the NMR analysis indicate that during acidification the Mg^2+^ ions are dissolved out of the Mg_2_Si_3_O_8_ and the material begins to dissolve and transition into a SiO_4_ tetrahedral structure with single and geminal silanol groups present on the outer surface. The acid treatment dissolves the material and perturbs both Si─O─Mg and Si─O─Si bonds, leading to structural changes and eventual dissolution of the RSP into silicic acid, SiOH_4_.

### 2.3 Amorphous SiO_2_ Recovery and Characterization

**Figure 6:**
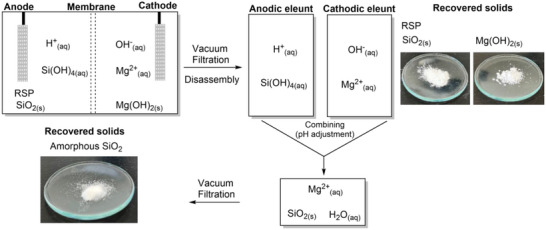
Schematic depiction of the electrolysis products obtained after disassembly of the electrolysis cell. Filtration of the anodic and cathodic compartments affords the residual silica phase (RSP) and Mg(OH)_2_ products, respectively. SiO_2_ can be recovered from the system via pH adjustment by combining the cathodic eluent with the anodic eluent containing dissolved Si(OH)_4_. Stoichiometric quantities are omitted for brevity.

Chronopotentiometry measurements conducted at longer time scales of 72 h under identical conditions results in nearly complete dissolution of the starting Mg_2_Si_3_O_8_ silicate material and the formation of quantitatively more Mg(OH)_2_. Interestingly, although there exists only trace amount of the RSP after longer electrolysis times, which itself is devoid of Mg^2+^ ions (**Figure** 5a), the yield of the Mg(OH)_2_ after 72 h remained low (≈40 %). This low yield can be ascribe to a multitude of facets governing the electrolyzer. We attribute this primarily to cell design and absence of mixture agitation (static conditions) within the cathodic compartment. Lack of adequate mixing can lead to local zones of extreme supersaturation specifically in our two compartment cell set‐up which favor rapid nucleation over crystal growth.^[^ ^34^ ^]^ Such rapid nucleation can result in impaired filtration and hinder solid‐liquid separation due to smaller particle sizes.^[^ ^35^ ^]^ Electrolyte choice may also play a role in Mg(OH)_2_ yield as an electrolyte containing cations which would co‐precipitate to form the analyte product (in this case Mg^2+^ ions) would substantially improve yields, although parasitic H^+^ migration into the cathodic compartment also affects Mg(OH)_2_ formation.^[^ ^7d^ ^]^ After 72 h of galvanostatic electrolysis the Mg(OH)_2_ formed in the cathodic compartment and minor amounts of RSP in the anodic compartment were filtered and removed. The eluent from the cathodic compartment was then added to the eluent from the anodic compartment (pH approx. 1.1, see **Figure** 1a) to increase to pH > 3. This pH adjustment resulted in the precipitation and polymerization of amorphous SiO_2_ which had dissolved in the acidic solution in the form of SiOH_4_, similar to sol‐gel synthesis methodology.^[^ ^36^ ^]^ The precipitated SiO_2_ was subsequently filtered, washed with deionized water and left to dry in the fume hood overnight. A schematic depiction of this process is shown in **Figure** **6**.

**Figure 7:**
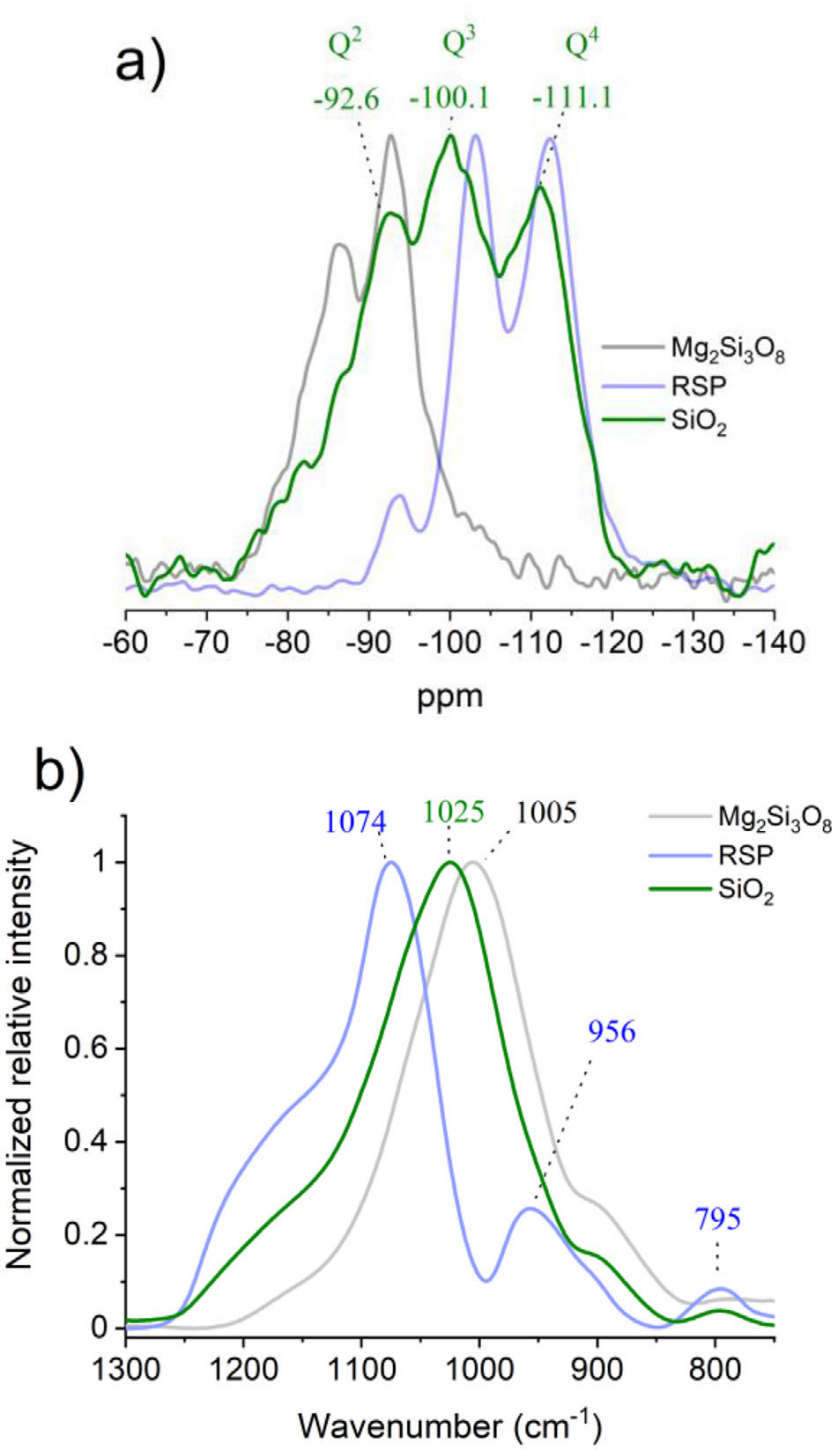
a) ^29^Si MAS NMR spectra b) FTIR spectra. Both depict amorphous SiO_2_ recovered after pH adjustment of anodic eluent (green traces); Mg_2_Si_3_O_8_ starting material (grey traces); Residual silica phase (RSP) after 24 h of electrolysis (light blue traces).

EDX analysis of the collected silica product confirms the formation of SiO_2_ however, impurities in the form of MgO are observed, likely due to residual Mg^2+^ ions dissolved in the cathodic eluent (Figure S13, Supporting Information). XRD analysis displays a large, broad band on the 2theta axis and indicates that the generated silica is indeed in the amorphous phase (Figure S14, Supporting Information),^[^ ^37^ ^]^ and SEM images display granular loosely‐packed particles (Figure S15, Supporting Information). The collected SiO_2_ was subsequently analyzed via ^29^Si NMR and is depicted in **Figure** **7**a (green trace), overlaid onto the previous spectra. The ^29^Si NMR spectrum of the SiO_2_ displays a broad signal centered at ≈−100 ppm with a full width at half maximum (FWHM) of 27.1 ppm (1618 Hz), consisting of three signals observed at −92.6, −100.1 and −111.1 ppm. The signal at −111.1 ppm is assigned as a Q^4^ group and is typical for fully polymerized amorphous SiO_2_, where SiO_4_ exists in a tetrahedral orientation.^[^ ^38^ ^]^ The signal observed at −100.1 ppm alludes to the presence of Q^3^ environments, attributed to surface Si─OH hydroxyl groups or other defects within the silica network.^[^ ^32b^ ^]^ The signal observed at ‐92.6 ppm is tentatively assigned to Si atoms in a Q^2^ environment, suggesting some amount of incomplete polymerization. The overall low resolution of the NMR peak indicates the final product is predominately disordered or in the amorphous phase, as the distribution of isotropic chemical shifts for each site broadens peak width.^[^ ^39^ ^]^ These resonances are in agreement with those observed for alumina‐free silica gels described by Neto and Skibsted which highlight the utility of this SiO_2_ material as an effective cement binder source.^[^ ^40^ ^]^ FTIR was conducted on the amorphous SiO_2_ and is depicted in Figure 7b. The spectrum is overlaid with the spectra of Mg_2_Si_3_O_8_ starting material (grey) and the RSP (light blue) after 24 h of electrolysis. The FTIR spectra in the region of 705‐1300 cm^−1^ constitutes the region where the Si─O─Si asymmetric stretching frequency is prominent, providing insight into the coordination environment of the materials,^[^ ^41^ ^]^ with the full spectra observable in Figure S16 (Supporting Information). The starting material Mg_2_Si_3_O_8_ displays one notable peak at 1005 cm^−1^, suggestive of the Mg^2+^ ions which affect the Si─O─Si bond frequency, shifting it to lower wavenumbers than typically observed for SiO_2_.^[^ ^42^ ^]^ The RSP collected after 24 h of electrolysis displays one large peak at 1074 cm^−1^ with two smaller peaks at 956 and 795 cm^−1^. The peak center at 1074 cm^−1^ can be interpreted as a higher degree of silicate polymerization devoid of the previous metal cations. The smaller peaks located at 956 and 795 cm^−1^ are indicative of the surface silanol groups and are attributed to the asymmetric Si─OH and symmetric Si─O─Si bending modes respectively,^[^ ^43^ ^]^ underscoring again the decomposition of the silicate material through acid digestion seen also in the NMR investigations. The amorphous SiO_2_ (green trace) exhibits one broad peak centered at 1025 cm^−1^, representative of amorphous SiO_2_, with lower internal order of the material generally leading to peak broadening.^[^ ^41^ ^]^

The NMR and FTIR spectra are in agreement and indicate that the Mg_2_Si_3_O_8_ is effectively dissolved in the acidic environment from continual proton production generated via water oxidation at the anode. This acid environment initially dissolves out the Mg^2+^ cations, causing condensation of residual silica fragments and substitution of these metal ions for H^+^. The protonation of the silica results in the formation of surface silanol groups, which, after extended periods, dissolve into solution in the form of SiOH_4_. Amorphous SiO_2_ was recovered from the system from dissolved SiOH_4_ by adjusting the pH, in this case through the addition of the solution generated in the cathodic compartment to the solution generated in the anodic compartment. This amorphous SiO_2_ collected holds utility as an effective binder source in M‐S‐H, as the formation of M‐S‐H cements rely substantially on the physio‐chemical properties of the precursors.

### 2.4 BET and Surface Area Analysis

**Figure 8:**
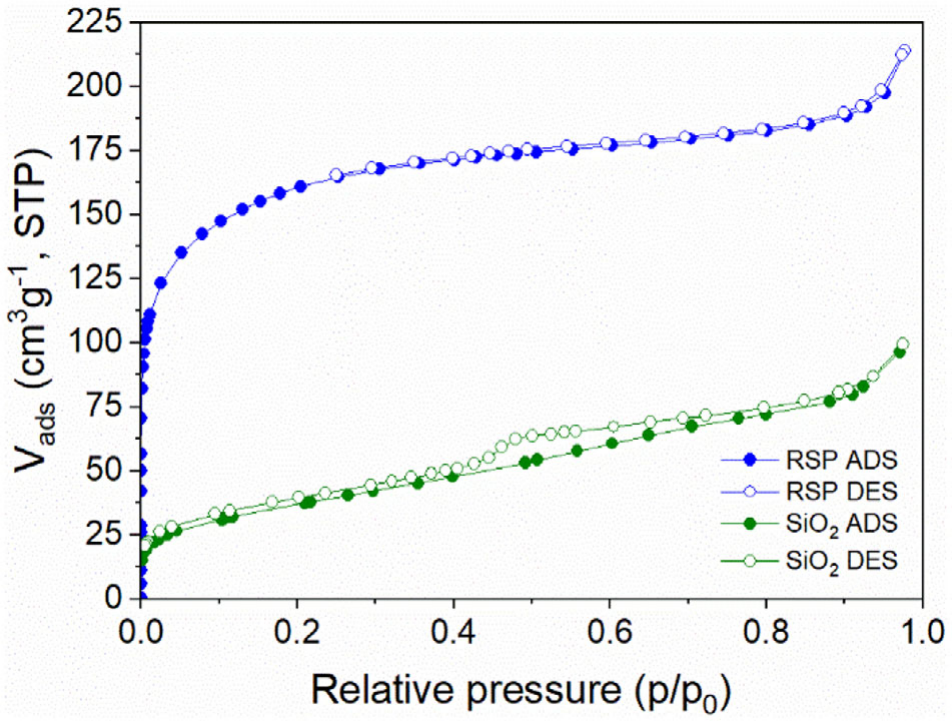
Nitrogen adsorption–desorption isotherm (ADS and DES, respectively) of the residual silicate phase (RSP) and amorphous SiO_2_ measured at 77 K, showing the volume of N_2_ adsorbed as a function of relative pressure (p/p₀). The isotherms were used to calculate the specific surface area of each sample.

BET (Brunaer‐Emmett‐Teller) analysis was conducted on the RSP and the recovered amorphous SiO_2_ using the N_2_ physisorption isotherms at 77 K, depicted in **Figure** **8**. From the isotherms, the specific surface areas of the materials were calculated. The RSP was found to be microporous, displaying an isotherm type I according to the IUPAC classification, with a surface area of 587 m^2^ g^−1^, calculated from p/p_0_ = 0.005 to 0.103, following the IUPAC guidelines,^[^ ^44^ ^]^ and total pore volume at of 0.331 cm^3^ g^−1^ at p/p_0_ = 0.98. The amorphous SiO_2_ was found to be mesoporous, isotherm type IV, with a surface area of 134 m^2^ g^−1^, calculated from p/p_0_ = 0.05 to 0.3, and total pore volume of 0.154 cm^3^ g^−1^ at p/p_0_ = 0.98. The RSP has a higher surface area compared to that of the amorphous SiO_2_, likely due to the proton passivation affecting porosity and increasing surface area. However, parameters such as pH, time and temperature can influence the surface area of SiO_2_ precipitated from Si(OH)_4_ in solution,^[^ ^45^ ^]^ suggesting the surface area of the recovered amorphous SiO_2_ can be modulated through such parameter adjustments. Pore size distributions for both samples were calculated via NLDFT cylinder pore model for N_2_ at 77 K and are depicted in Figures S17 and S18 (Supporting Information). The RSP presents a representative amount of micropores at 0.43, 0.8, and 1.8 nm, with 54% of its cumulative pore volume below 2 nm, while SiO_2_ shows 70% of its pore volume between 2 and 5 nm, with a distinct peak at 5 nm.

Importantly, both the RSP and amorphous SiO_2_ have higher surface areas than typical silica fume (≈30 m^2^ g^−1^).^[^ ^46^ ^]^ High surface area SiO_2_ has implications in rheology control and can result in disperse cement particles, reducing yield stress and enhancing workability.^[^ ^47^ ^]^ Silica with a higher degree of amorphicity and larger surface area have been shown to improve the rate of silica dissolution, enhancing M‐S‐H formation.^[^ ^28^, ^48^ ^]^

## 3 Conclusion 

The synthetic silicate Mg_2_Si_3_O_8_ can be transformed into the cementitious precursors Mg(OH)_2_ and amorphous SiO_2_ as a result of water electrolysis at ambient conditions to generate H^+^, which dissolves the silicate material and OH^−^ to which the Mg^2+^ ions coordinate. pH and conductivity measurements depict the dissolution of the material over 24 h and illustrate how the electrolysis of water lowers the pH to effectively dissolve the Mg_2_Si_3_O_8_ into its constituent components. XRD, NMR and SEM analysis indicate the dissolution of the Mg_2_Si_3_O_8_ initially results in the removal of the Mg^2+^ ions from the material and produces a partially ordered SiO_2_ composite (RSP) with hydroxylated end groups before dissolution to Si(OH)_4_. In the cathodic compartment, the generated Mg(OH)_2_ is observed to organize into large, platelet‐like structures over 500 µm in length. SiO_2_ can be recovered from the system and NMR, IR and XRD spectroscopic analysis demonstrates that the SiO_2_ is predominately in the amorphous phase. The dissolution of Mg_2_Si_3_O_8_ utilizes the same underlying principles of electrochemical cement precursor production using carbonate minerals, with the caveat that no CO_2_ is produced in the dissolution of the starting material, alleviating the need for energy intensive carbon capture and transformation.

## 4 Implications in Cement Manufacturing

Although the Mg_2_Si_3_O_8_ is a synthetic material, the dissolution of ultramafic minerals such as olivine and fosterite display dissolution mechanisms similar to this synthetic material, wherein protonation of the minerals results in exchange of the Mg^2+^ ions with H^+^ ions at low pH.^[^ ^49^ ^]^ The amorphous nature of the Mg_2_Si_3_O_8_ may result in faster decomposition rates than its natural mineral counterparts, however the lower Mg:Si ratio of the Mg_2_Si_3_O_8_ may form larger quantities of hydrated silica (H_x_Si_y_O_z_), impeding dissolution and requiring longer reactions time. Further kinetic investigations are necessary for a quantitative comparison of dissolution rates. Nevertheless, the synthetic Mg_2_Si_2_O_3_ materials represents an adequate model system for silicate mineral transformation utilizing an electrolyzer. The amorphous silica produced as a byproduct of the reaction, either the RSP or the amorphous SiO_2_ collected after pH adjustment represents and avenue for generating amorphous and high surface area SiO_2_, circumventing the need for outside sources of SiO_2_ in cement manufacturing. However, although the RSP collected has a higher surface area compared to the amorphous SiO_2_ (585 vs 134 m^2^ g^−1^) respectively, too high of surface area may lead to yield stress in the resultant cement workability.^[^ ^50^ ^]^ Nevertheless, the surface are can be influence by processing parameters (longer dissolution times, pH adjustments) and could therefore be tailored or adjusted. The amorphous SiO_2_ collected can likely function as an effective binder alternative source in the component mixture process for cement, typically performing better than other silicate sources due to its high degree of amorphicity and surface area.^[^ ^13^, ^48^ ^]^

This methodology can be coupled with effective separation techniques of metal ions or other components within mineral phases and extended to complex earth‐abundant silicate minerals containing metal ions (olivine, serpentine, fosterite) or calcium (wollastonite) or mixtures of calcium and magnesium (pyroxenes), broadening the scope of feedstock minerals not only for M‐S‐H but also Ca‐based cement production. The energy requirements have been outlined elsewhere and suggest this electrolysis method can have practicality at large scales.^[^ ^6^ ^]^ Juxtaposed to conventional cement manufacturing, this electrochemical manufacturing method allows for the utilization of abundant silica‐based materials or mining waste and their transformation utilizing electricity eradicating the production of CO_2_ in the transformation of the material. This methodology represents a promising pathway for mineral transformation of unconventional starting materials for cement, CO_2_ abatement and sustainable energy production in the form of H_2_ and O_2_ gases.^[^ ^51^ ^]^ If the generated gas streams are utilized for calcination, the entire cement manufacturing process can in principle be conducted without the production of CO_2_ or other waste products from fossil fuel ignition. When powered by renewable energy sources, if CO_2_ is incorporated during the carbonation of M‐S‐H cement, the electrochemical approach can be effectively carbon negative. Ideally, this electrochemical approach could transform the paradigm in which magnesium or traditional calcium‐based cements are produced. At the present moment, the current system is small and likely faces engineering complications in the face of scalability. However, the utility of this system should not be overshadowed, and although synthetic, Mg_2_Si_3_O_8_ represents a step toward large scale cement production using prevalent siliceous minerals. Such investigations are currently underway in our laboratory.

## Conflict of Interest

The authors declare no conflict of interest.

## Supporting information



Supporting Information

## Data Availability

The data that support the findings of this study are openly available in Zenodo at https://doi.org/10.5281/zenodo.15797981, reference number 15797981.
