# Supplementary material for: Transformation of a Silicate Material for Carbon Negative Magnesia‐Based Cement via Electrochemistry
Source: Adv Sci (Weinh). 2025 Aug 20;12(42):e04141. doi: 10.1002/advs.202504141 (PMC12622475; doi:10.1002/advs.202504141)
Supplement: Supplementary file 1 — Supporting Information [file ADVS-12-e04141-s001.docx]

*Supplementary Information for*

**Transformation of a silicate material for carbon negative magnesia-based cement via electrochemistry**

Anthony R. Ramuglia,^a*^ Julius Scheel,^a^ Thomas Köberle,^a^ Kelly Henze,^b^ Silvia Paasch,^c^ Lairana Lima Duarte,^d^ Stefan Kaskel,^d^ Eike Brunner,^c^ Viktor Mechtcherine,^a^ Thomas Matschei,^e^ Inez. M. Weidinger^f*^ and Marco Liebscher^a*^

[a] Dr. A.R. Ramuglia, J. Scheel, Dr. T. Köberle, Prof. Dr. V. Mechtcherine, Prof. Dr. M. Liebscher
Institute of Construction Materials
Technische Universität Dresden
Georg-Schumann-Straße 7, 01187 Dresden
E-mail: [anthony.ramuglia@tu-dresden.de](mailto:anthony.ramuglia@tu-dresden.de), [marco.liebscher@tu-dresden.de](mailto:marco.liebscher@tu-dresden.de)

[b] K. Henze
Chair of Physical Chemistry, Faculty of Chemistry and Food Chemistry
Technische Universität Dresden
Zellescher Weg 19, 01069 Dresden (Germany)

[c] Dr. S. Paasch, Prof. Dr. E. Brunner
Chair of Bioanalytical Chemistry, Faculty of Chemistry and Food Chemistry
Technische Universität Dresden
Bergstraße 66, 01069 Dresden (Germany)

[d] L. Duarte, Prof. Dr. Stefan Kaskel
Boysen-TU Dresden-Research Training Group

TUD Dresden University of Technology
Chemnitzer Str. 48B, 01187 Dresden

&
Bergstraße 66, 01069 Dresden (Germany) Chair of Inorganic Chemistry I, Faculty of Chemistry and Food Chemistry
Technische Universität Dresden
Bergstraße 66, 01069 Dresden (Germany)

[e] Prof. Dr. T. Matchei
Institute of Building materials research and Chair of Building Materials
Aachen University
Schinkelstrasse 3, 52062 Aachen (Germany)

[f] Prof. Dr. I.M. Weidinger
Chair Electrochemistry, Faculty of Chemistry and Food Chemistry
Technische Universität Dresden
Zellescher Weg 19, 01069 Dresden (Germany) E-mail:
E-mail: [Inez.Weidinger@tu-dresden.de](mailto:Inez.Weidinger@tu-dresden.de)

Table of Contents

[Materials and Methods 3](#_Toc202457944)

[Electrochemical measurements 5](#_Toc202457945)

[Electrolyzer efficiency 7](#_Toc202457946)

[pH and conductivity measurements 8](#_Toc202457947)

[Mg(OH)_2_, RSP and amorphous SiO_2_ characterization 12](#_Toc202457948)

[Substrate Characterization 18](#_Toc202457949)

[Electrolysis set-up and electrode characterization 20](#_Toc202457950)

[References: 23](#_Toc202457951)

# Materials and Methods

Magnesium trisilicate referred to here as Mg_2_Si_3_O_8_ was used as the starting material in every experiment. Unless otherwise stated the supporting electrolyte was composed of 0.5 M sodium sulfate for all experiments with the exception of one using KNO_3_. The Mg_2_Si_3_O_8_ and electrolyte, sodium sulfate decahydrate (Na_2_SO_4_ ⋅ 10H_2_O) (≥ 99%) were purchased through Roth chemical and the potassium nitrate (KNO_3_) was purchased from Sigma Aldrich. Each chemical was used as received. Milli Q deionized water with a conductance of 0.055 μS was used for all experiments. The Mg_2_Si_3_O_8_ starting material itself was analyzed via EDX, XRD and Raman spectroscopy in addition to the characterization techniques displayed in the main text. These figures can be found in the Substrate Characterization section on page 18 and 19 Figures 19-21.

Electrolysis experiments were conducted using 1.0 g (3.82 x 10^-3^) moles of the Mg_2_Si_3_O_8_ starting material at room temperature. A Pt mesh electrode on Ti was utilized for both the cathode and anode, both measuring approximately 30 mm x 35 mm, with electroactive surface area of 9.0 +/- 0.5 cm^2^, calculated through the integration of hydrogen adsorption on Pt in 0.5 M H_2_SO_4_ (page 15 Figure 14). An Ag/AgCl electrode in 3 M KCl solution separated by a glass frit constituted the reference electrode for all electrolysis measurements. LSV measurements conducted with a Hydoflex RHE (reversible hydrogen electrode) purchased from gaskatel.

Electrochemical experiments were conducted using a Biologic SP-300 potentiostat with EC-Lab software and Ivium Vertex One EIS potentiostat with Ivium electrochemical software. All electrochemical measurements were performed in a two compartment “H-cell” containing a total volume of 140 mL, consisting of 70 mL of supporting electrolyte in each compartment. The two compartments were partitioned with a Nafion 115 cation exchange membrane. The membrane was activated through first treatment in 3 wt % H_2_O_2_ for one hour at 80 °C followed by treatment in deionized water at 80 °C for one hour, followed by subsequent treatment in 0.5 M H_2_SO_4_ at 80 °C for one hour and finally rinsed and stored in deionized water at room temperature. Pictures of the electrochemical setup can be found on page 16, Figures 15-17.

pH and conductivity measurements were conducted on an APERA820 pH and conductivity meter. and were recorded every 30 min during electrolysis, with the applied current pulsed from to open circuit potential (OCP) every 30 min with pH and conductivity measurements taken every 31 min to allow for one minute equilibration time.

XRD measurements were performed on a Bruker 2D phaser in a 2θ range of 5 - 90° for 2.5 h. Samples were dispersed in ethanol, drop-cast on a Si wafer and dried in ambient air.

SEM images were taken on a ESEM Quanta FEG250 (FEI) with connected EDX QUANTAX 400 (Bruker).

EDX measurements were performed on the respective powder at magnification 2000x (frame: 200 x 140 µm) with 20kV and spot size 4.0, with 5 measurements carried out on each sample.

^29^Si MAS NMR experiments were recorded on a Bruker Avance 300 spectrometer operating at 59.62 MHz using a commercial double resonance 4 mm MAS NMR probe. SPINAL ^1^H decoupling was applied during signal acquisition. The MAS frequency was 10 kHz for all samples. The recycle delay was 90 s and DSS (Sodium trimethylsilylpropanesulfonate) was utilized as external standard.

Raman spectroscopy was conducted using a confocal Raman microscope (S&I Monovista CRS+) with laser excitation by a Hubner Photonics Cobolt 04-01 series single frequency 514 nm diode laser. The spectra were referenced to toluene as a standard.

FTIR spectroscopy was conducted using a Bruker Tensor II infrared spectrometer with diamond ATR collection head at wavenumbers between 675 and 4000 cm^-1^.

BET isotherms were measured using Belsorp Max II, and Quadrasorp using N_2_ gas at 77 K. All samples were degassed for 12 hours at 150°C under vacuum prior to the measurement.

# Electrochemical measurements


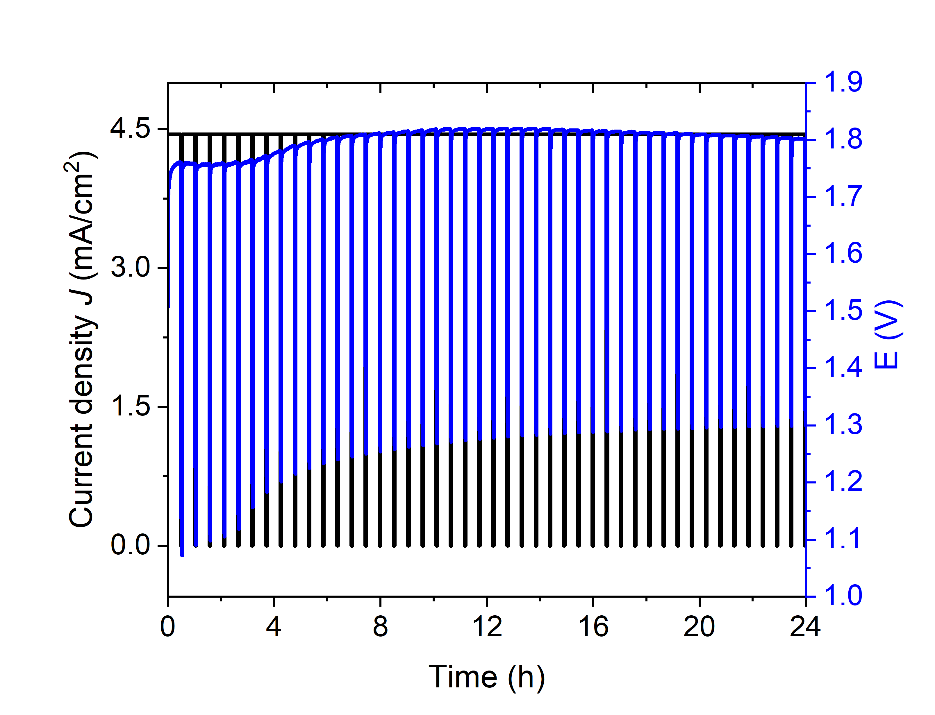


Figure 1. Pulsed chronopotentiometry conducted at 40 mA. The current was applied for 30 min before switching to open circuit potential (OCP) for a 2 min increment, repeated for 24 hrs.


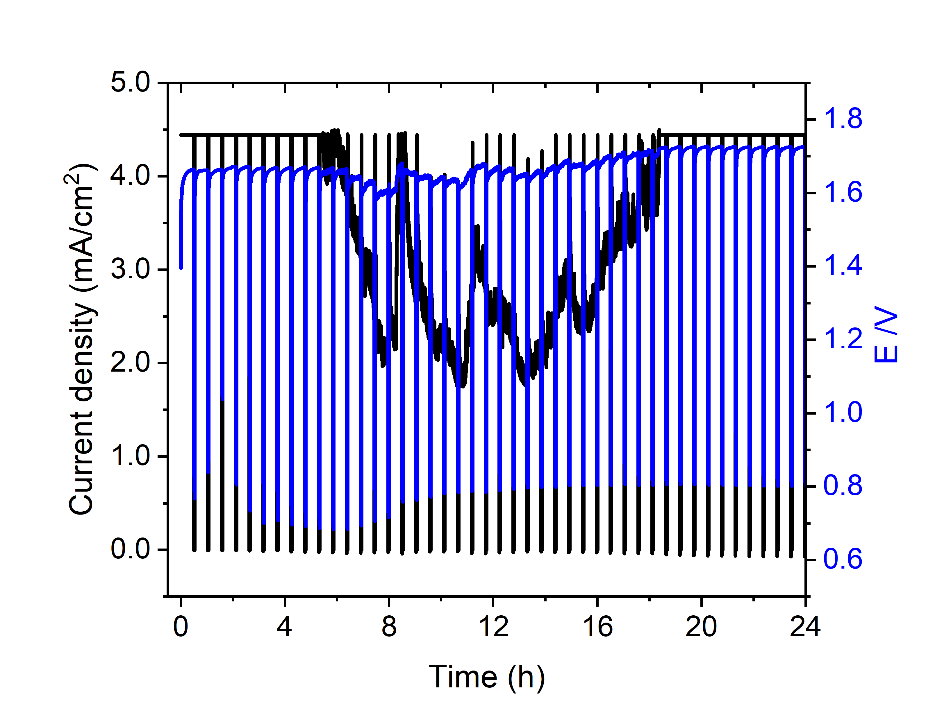


Figure 2. Pulsed chronopotentiometry conducted at 40 mA. The current was applied for 30 min before switching to open circuit potential (OCP) for a 2 min increment repeated for 24 hrs. The current density is observed to attenuate over the course of the electrolysis, likely due to membrane passivation.


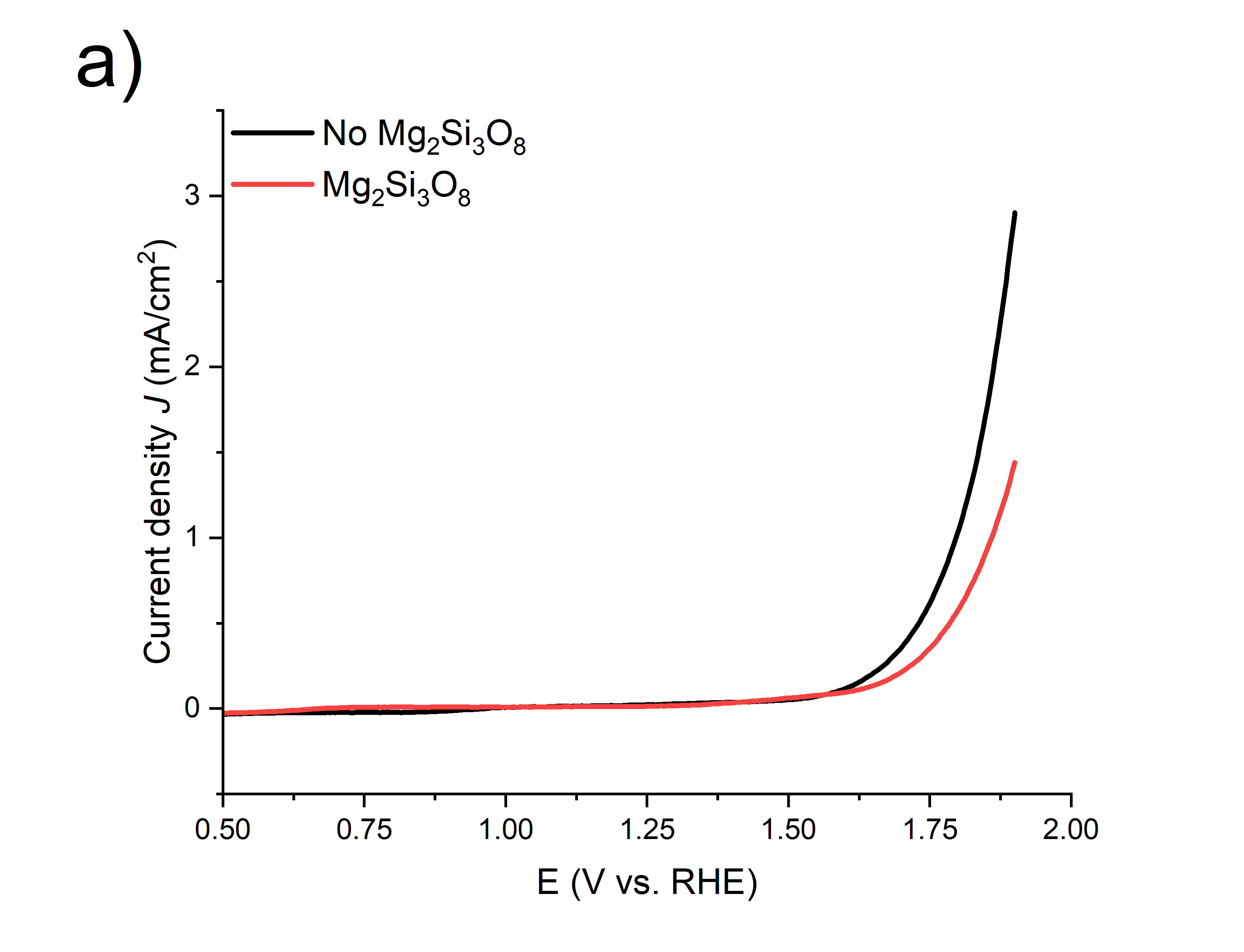

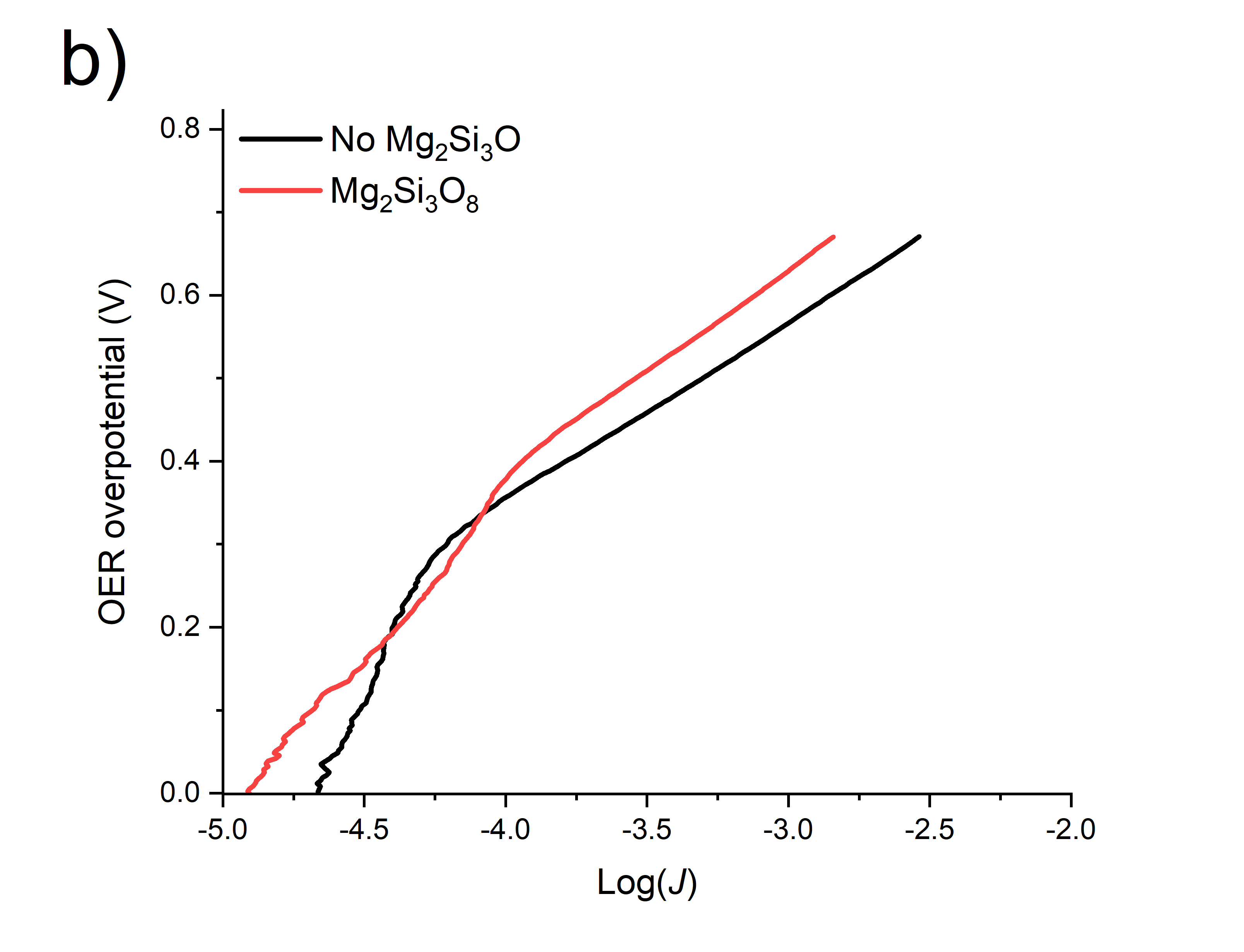


Figure 3. a) Linear sweep voltammetry (LSV) of the system in the presence and absence of Mg_2_Si_3_O_8_, vs. reversible hydrogen electrode RHE at 1mV/s with 85% *i*R correction conducted by the potentiostat during data collection. b) Tafel plots extrapolated from the LSV measurements. Tafel slopes calculated to be 320 mV/dec and 290 mV/dec in the presence and absence of Mg_2_Si_3_O_8_, respectively.


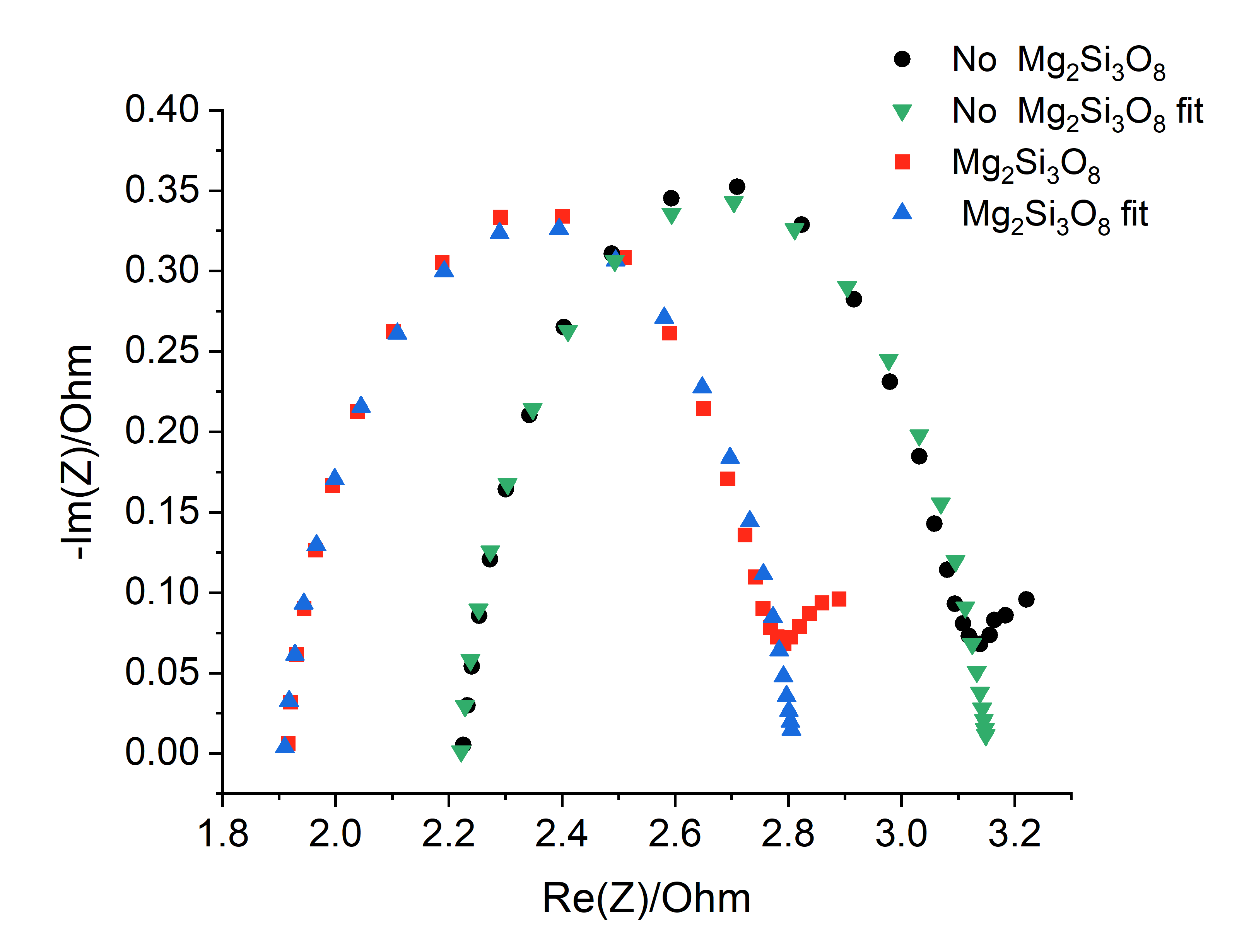


Figure 4. Galvanostatic electrochemical impedance spectroscopy (EIS) conducted at 40 mA with 4 mA amplitude from 100 kHz to 10 mHz. Fits were performed using EC-lab software with Monte-Carlo simulations fitting for the R1+R2/Q1 equivalent circuit.

# Electrolyzer efficiency

Figure 5. Gas Chromatograph of the gaseous products collected at the cathode during galvanostatic electrolysis at 40 mA

Table 1. Gas chromatograph result analysis of gaseous products collected at the cathode during galvanostatic electrolysis at 40 mA.

| Peak | Peak Name | Ret.Time (min) | Rel.Area | Area mV*min | Height mV |
| --- | --- | --- | --- | --- | --- |
| FrontDetector | FrontDetector | FrontDetector | FrontDetector | FrontDetector | FrontDetector |
| 1 | H2 | 5.862 | 79.03 | 4.8149 | 31.27 |
| 2 | O2 | 6.28 | 5.31 | 0.3234 | 3.93 |
| 3 | N2 | 6.783 | 15.66 | 0.9544 | 10.52 |
| Maximum |  |  | 79.03 | 4.8149 | 31.27 |
| Minimum |  |  | 5.31 | 0.3234 | 3.93 |
| Sum |  |  | 100 | 6.0927 | 45.72 |

Galvanostatic electrolysis was conducted at 40 mA. After 1114 seconds, 5 mL of gas was collected from the cathode of the electrolyzer at standard temperature and pressure. The resultant faradaic efficiency (FE) is approximately 76 %.

# pH and conductivity measurements

Table 2. Recorded pH values in the anodic compartment at each time interval (hrs) during galvanostatic electrolysis at 40 mA/cm^2^ in the presence of the starting material, Mg_2_Si_3_O_8_, at standard temperature and pressure. Y-error refers to standard deviation between each measurement.

| Time (hrs) | | Trial 1  pH | | Trial 2  pH | | Trial 3  pH | | Average  pH | Y-error  (Standard deviation) |
| --- | --- | --- | --- | --- | --- | --- | --- | --- | --- |
| 0 | | 8.68 | | 8.90 | | 8.97 | | 8.85 | 0.12 |
| 0.5 | | 7.35 | | 7.28 | | 7.03 | | 7.22 | 0.14 |
| 1 | | 6.78 | | 6.77 | | 6.62 | | 6.72 | 0.07 |
| 1.5 | | 6.50 | | 6.45 | | 6.37 | | 6.44 | 0.05 |
| 2 | | 6.13 | | 6.20 | | 6.18 | | 6.17 | 0.03 |
| 2.5 | | 5.89 | | 5.94 | | 6.00 | | 5.94 | 0.05 |
| 3 | | 5.95 | | 5.65 | | 5.82 | | 5.81 | 0.12 |
| 3.5 | | 5.84 | | 5.23 | | 5.62 | | 5.57 | 0.25 |
| 4 | | 5.53 | | 4.41 | | 5.37 | | 5.11 | 0.49 |
| 4.5 | | 5.26 | | 3.56 | | 5.07 | | 4.63 | 0.76 |
| 5 | | 4.76 | | 3.09 | | 4.64 | | 4.16 | 0.76 |
| 5.5 | | 3.54 | | 2.79 | | 4.09 | | 3.47 | 0.53 |
| 6 | | 3.58 | | 2.57 | | 3.51 | | 3.22 | 0.46 |
| 6.5 | | 3.01 | | 2.41 | | 3.10 | | 2.84 | 0.31 |
| 7 | | 2.70 | | 2.30 | | 2.82 | | 2.60 | 0.22 |
| 7.5 | | 2.63 | | 2.20 | | 2.61 | | 2.48 | 0.20 |
| 8 | | 2.45 | | 2.12 | | 2.45 | | 2.34 | 0.16 |
| 8.5 | | 2.29 | | 2.04 | | 2.32 | | 2.22 | 0.12 |
| 9 | | 2.21 | | 1.98 | | 2.22 | | 2.14 | 0.11 |
| 9.5 | | 2.12 | | 1.92 | | 2.13 | | 2.06 | 0.10 |
| 10 | | 2.10 | | 1.88 | | 2.06 | | 2.01 | 0.10 |
| 10.5 | | 2.02 | | 1.83 | | 2.00 | | 1.95 | 0.08 |
| 11 | | 1.95 | | 1.79 | | 1.93 | | 1.89 | 0.07 |
| 11.5 | | 1.89 | | 1.75 | | 1.88 | | 1.84 | 0.06 |
| 12 | | 1.82 | | 1.71 | | 1.84 | | 1.79 | 0.05 |
| 24 | | 1.11 | | 1.32 | | 1.39 | | 1.27 | 0.12 |
|  |  | |  | |  | |  |  |  |


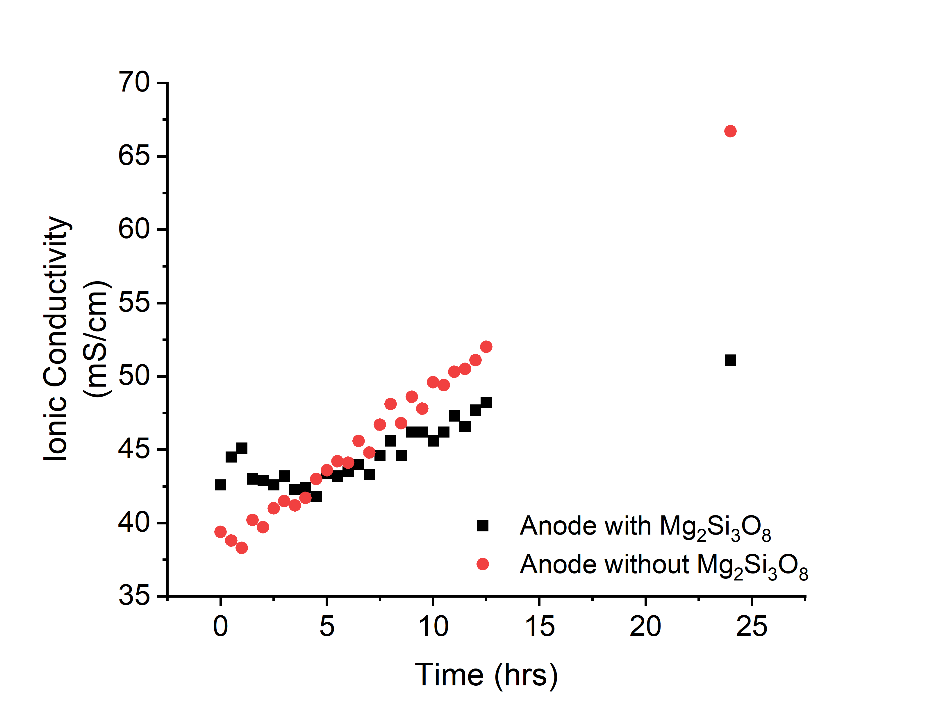


Figure 6. Ionic conductivity measurements during galvanostatic electrolysis at 40 mA in the anodic compartment in the presence and absence of the starting material (Mg_2_Si_3_O_8_) black and red traces, respectively. The difference in ionic conductivity at time zero is attributed to some small amount of dissolution of the Mg_2_Si_3_O_8_. Although the K_sp_ is not known, we assume it is small but nevertheless nonzero.

Table 3. Recorded ionic conductivity values in the anodic compartment at each time interval (hrs) during galvanostatic electrolysis at 40 mA/cm^2^ in the presence and absence of the starting material (Mg_2_Si_3_O_8_) at standard temperature and pressure.

|  | Conductivity at anode with Mg_2_Si_3_O_8_ | Conductivity at anode without Mg_2_Si_3_O_8_ |
| --- | --- | --- |
| Time (hrs) | mS/cm | mS/cm |
| 0 | 42.6 | 39.4 |
| 0.5 | 44.5 | 38.8 |
| 1 | 45.1 | 38.3 |
| 1.5 | 43 | 40.2 |
| 2 | 42.9 | 39.7 |
| 2.5 | 42.6 | 41 |
| 3 | 43.2 | 41.5 |
| 3.5 | 42.3 | 41.2 |
| 4 | 42.4 | 41.7 |
| 4.5 | 41.8 | 43 |
| 5 | 43.4 | 43.6 |
| 5.5 | 43.2 | 44.2 |
| 6 | 43.5 | 44.1 |
| 6.5 | 44 | 45.6 |
| 7 | 43.3 | 44.8 |
| 7.5 | 44.6 | 46.7 |
| 8 | 45.6 | 48.1 |
| 8.5 | 44.6 | 46.8 |
| 9 | 46.2 | 48.6 |
| 9.5 | 46.2 | 47.8 |
| 10 | 45.6 | 49.6 |
| 10.5 | 46.2 | 49.4 |
| 11 | 47.3 | 50.3 |
| 11.5 | 46.6 | 50.5 |
| 12 | 47.7 | 51.1 |
| 24 | 51.1 | 66.7 |


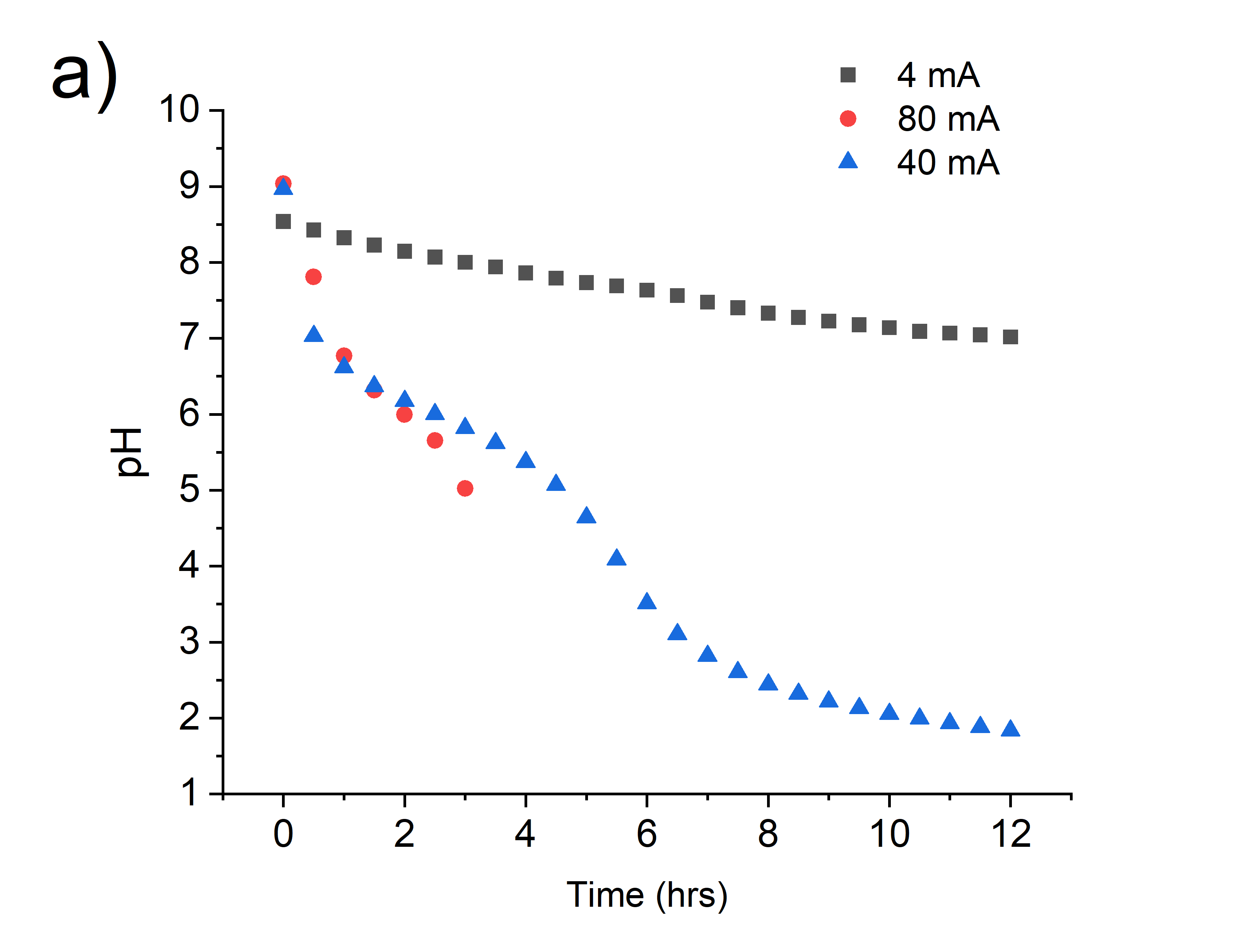

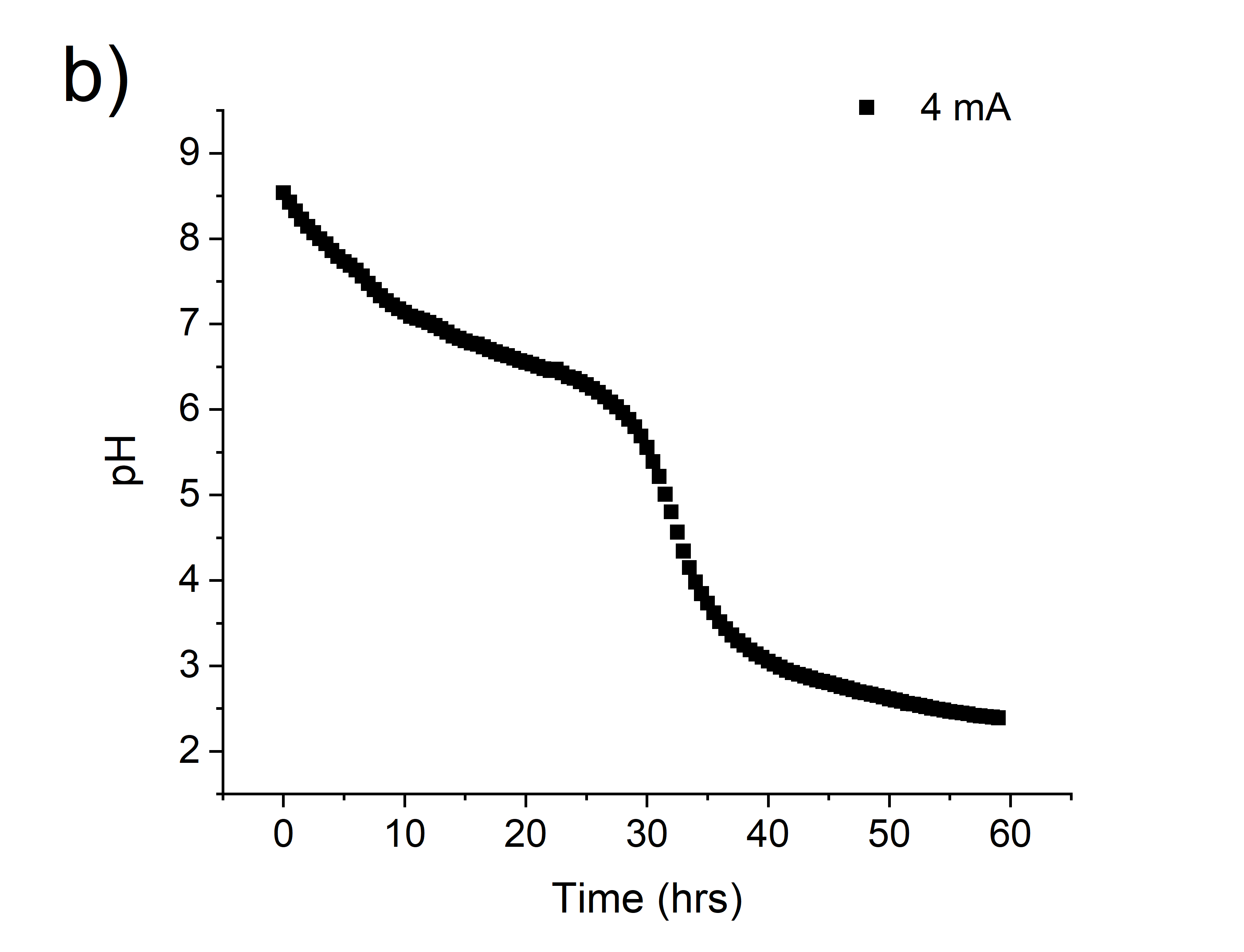


Figure 7. a) pH measurements during galvanostatic electrolysis at different applied currents. After 4 hrs at 80 mA the potentiostat experienced an amplifier overload error, resulting in low integrity data beyond this time point. b) pH measurements during galvanostatic electrolysis at 4 mA over longer timescales, displaying a similar trend compared to measurements at higher amperage.


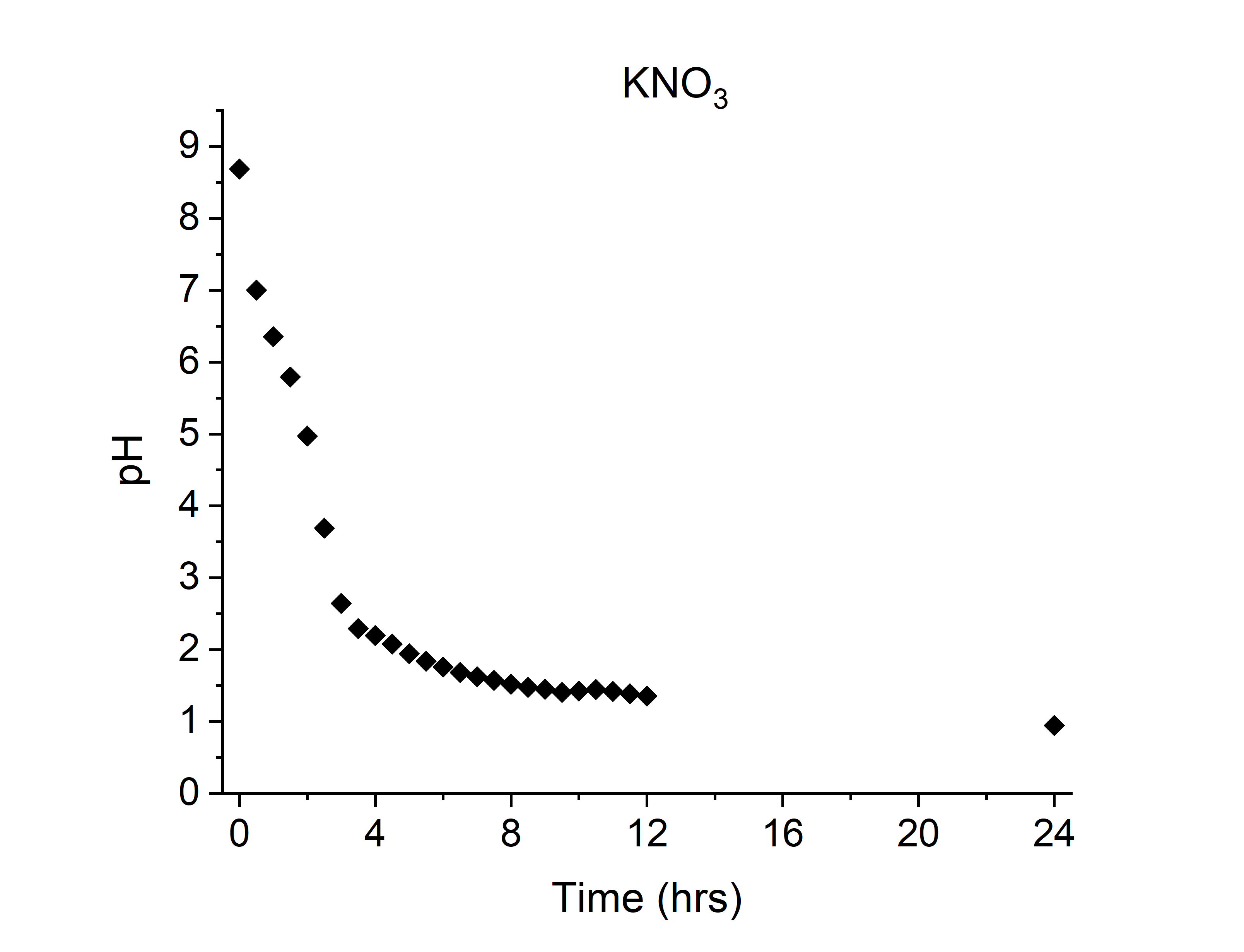


Figure 8. pH measurements during galvanostatic electrolysis at 40 mA in 0.5 M KNO_3_

# Mg(OH)_2_, RSP and amorphous SiO_2_ characterization


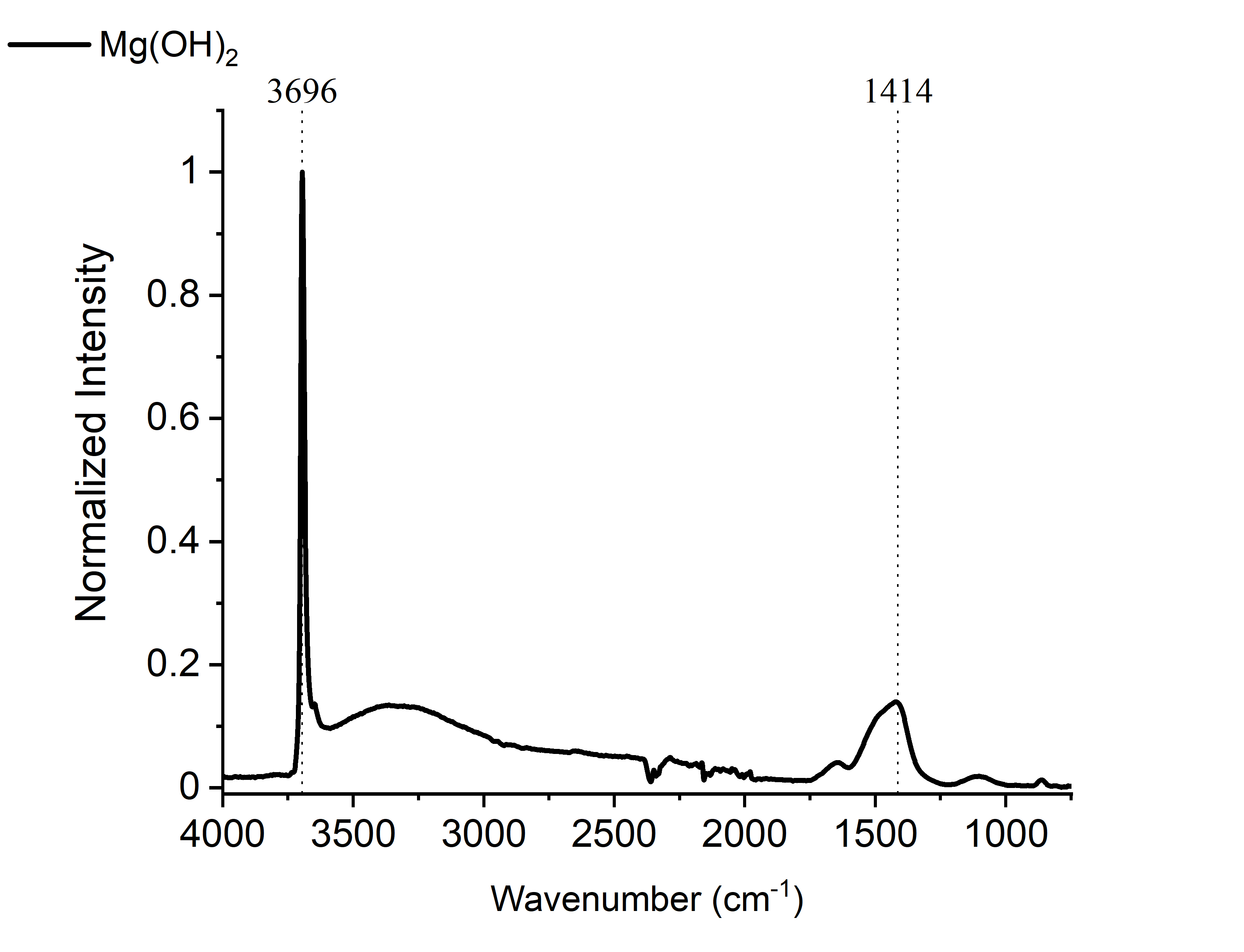


Figure 9. FTIR ATR spectrum of Mg(OH)_2_. The peak at 3696 cm^-1^ is attributed to the O—H stretching vibration^1, 2^, while the peak at 1414 cm^-1^ is a result of some amount of carbonation of the sample occurring during handling. The 1414 cm^-1^ is assigned to the CO_3_^2-^ vibrational mode of the Mg(CO)_3_.^3^


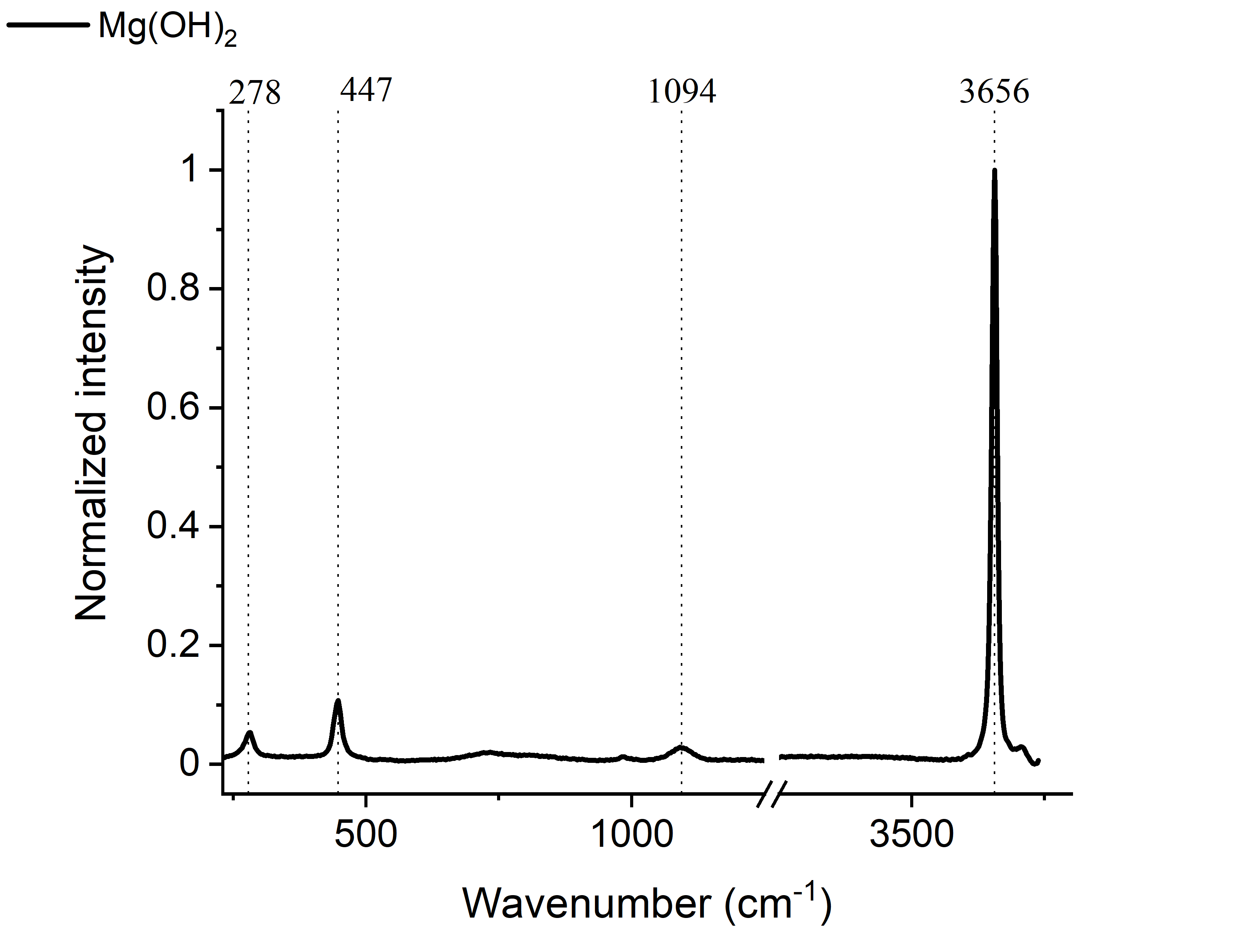


Figure 10. Raman spectrum at 514 nm excitation wavelength of Mg(OH)_2_. The band observed at 3656 cm^-1^ is attributed to the O—H stretching vibration and the bands at 278 and 447 cm^-1^ are attributed to the Mg—O vibrations.^4^ The band observed at 1094 cm^-1^ is similarly an artifact of carbonation occurring during sample handling and is assigned to the CO_3_^2-^ vibrational mode of the Mg(CO)_3_.^5^


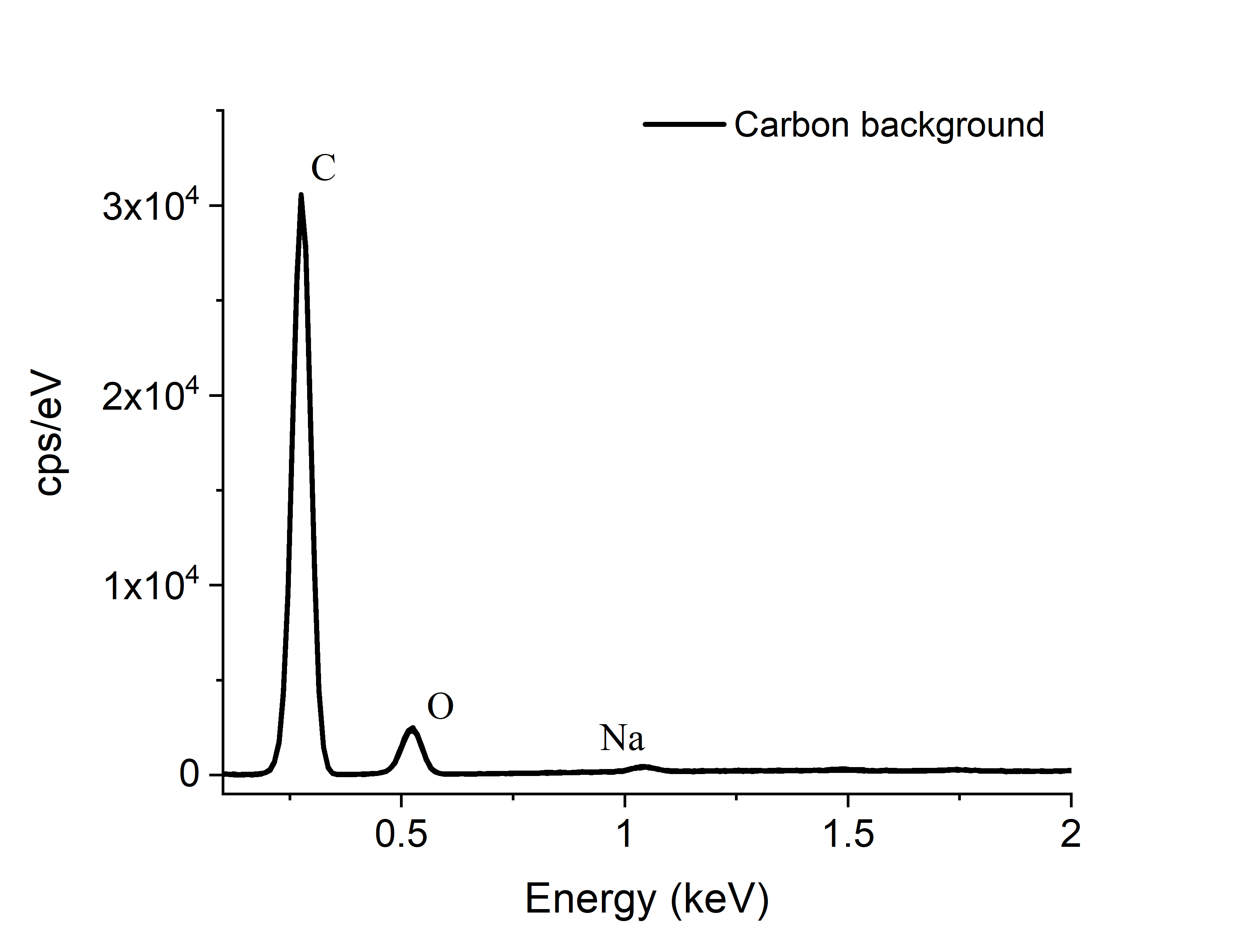


Figure 11. EDX spectrum of the carbon background onto which the sample was attached


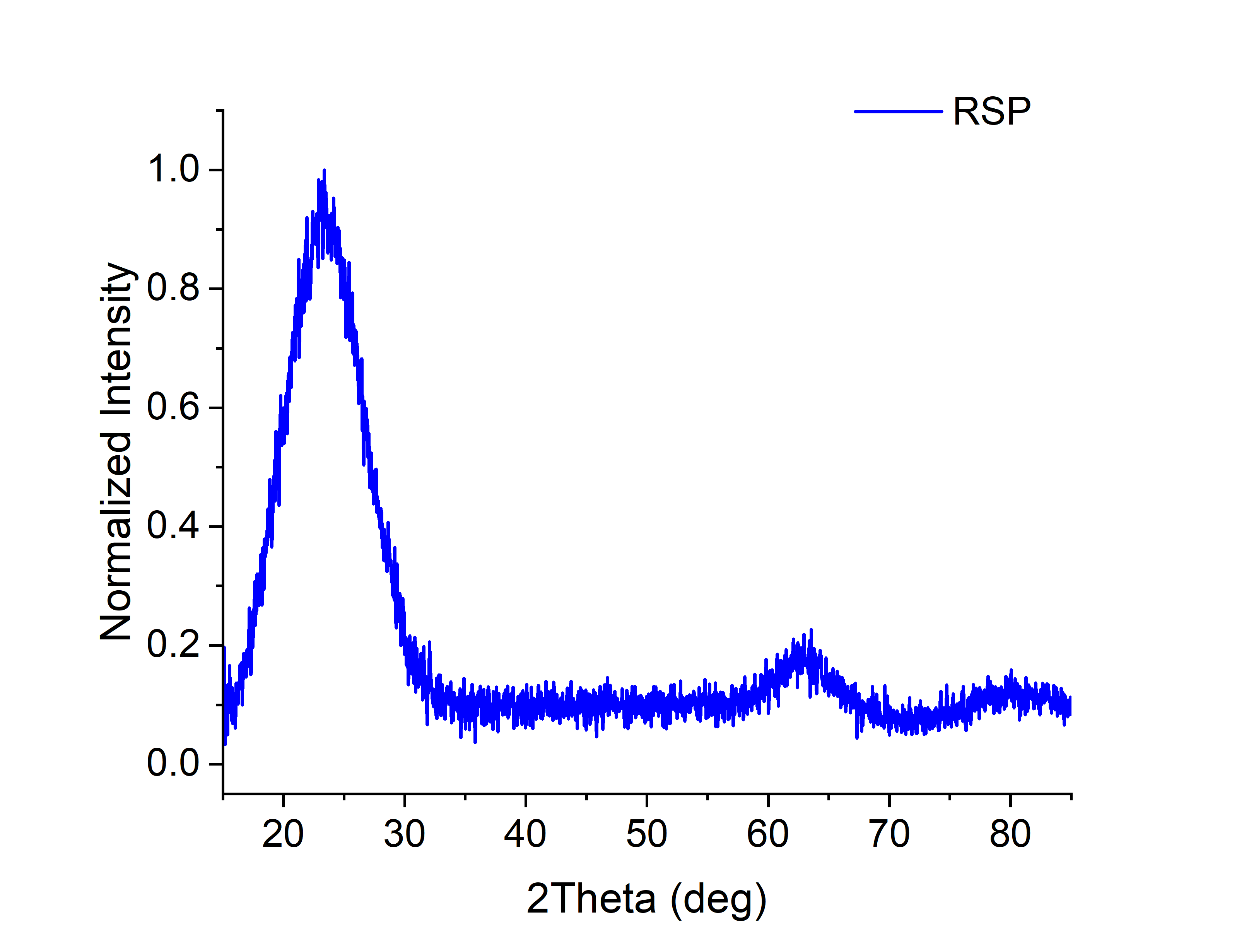


Figure 12. XRD spectrum of the RSP collected after 24 hrs electrolysis.


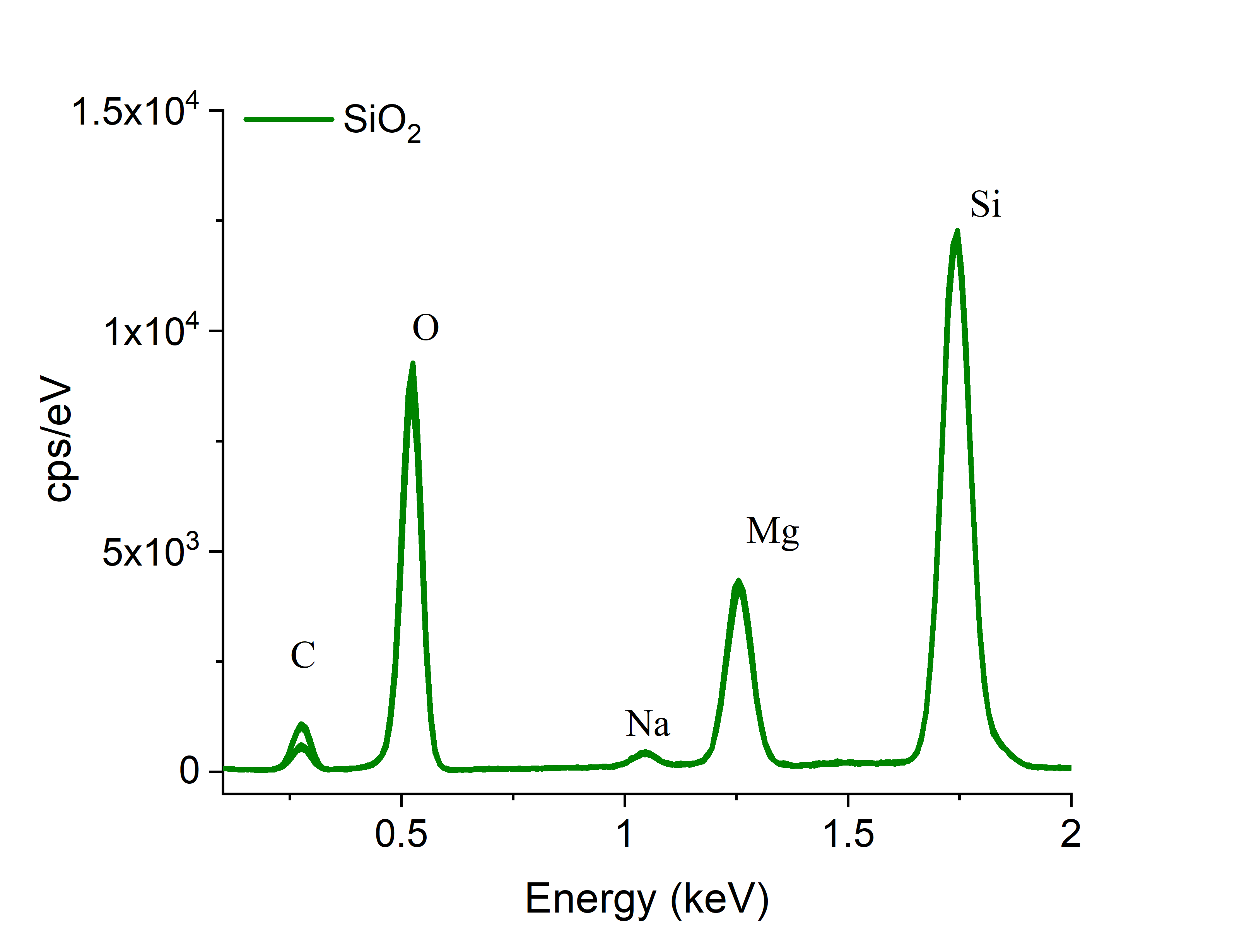


Figure 13. EDX spectrum of amorphous SiO_2_ recovered from the anodic eluent via pH adjustment to pH >3 using the cathodic eluent. The Mg peak is a result of minor amounts of Mg^2+^ ions left in in the cathodic eluent.


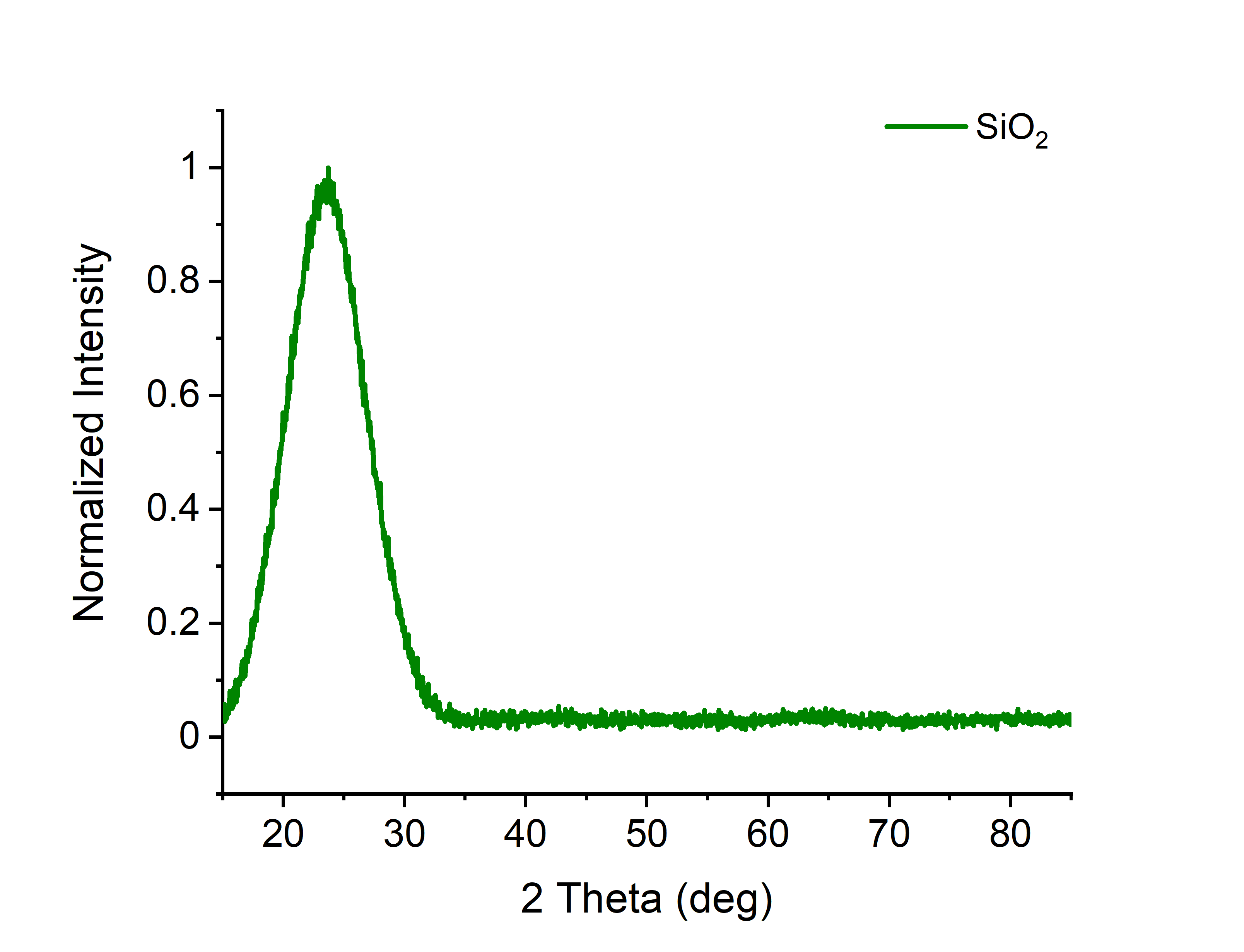


Figure 14. XRD spectrum of amorphous SiO_2_ recovered from the anodic eluent via pH adjustment to pH >3 using the cathodic eluent


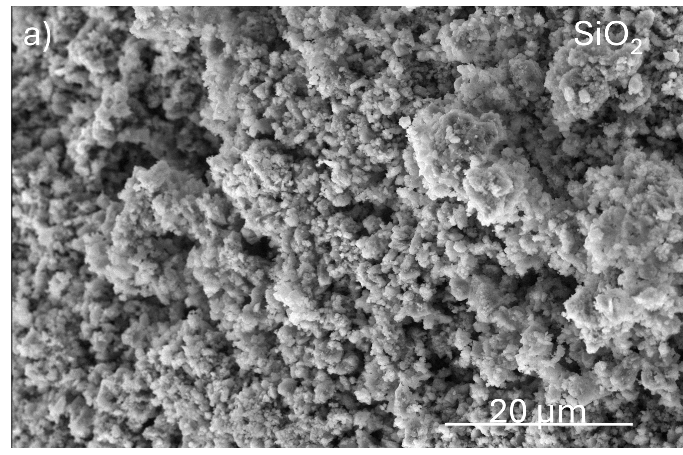

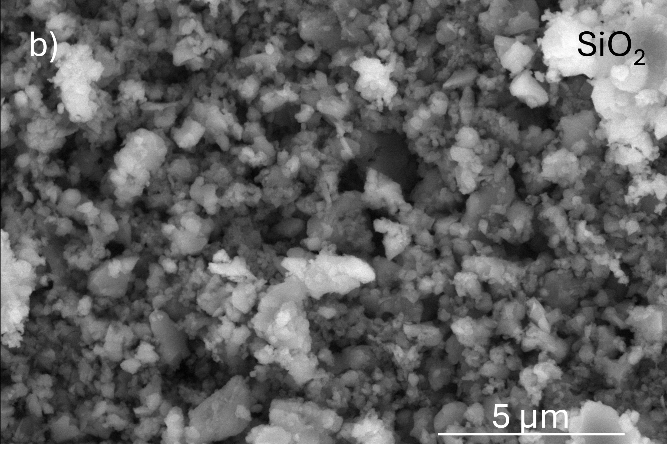


Figure 15. SEM images of amorphous SiO_2_. Left, 6000x magnification; Right 2400 x magnification.


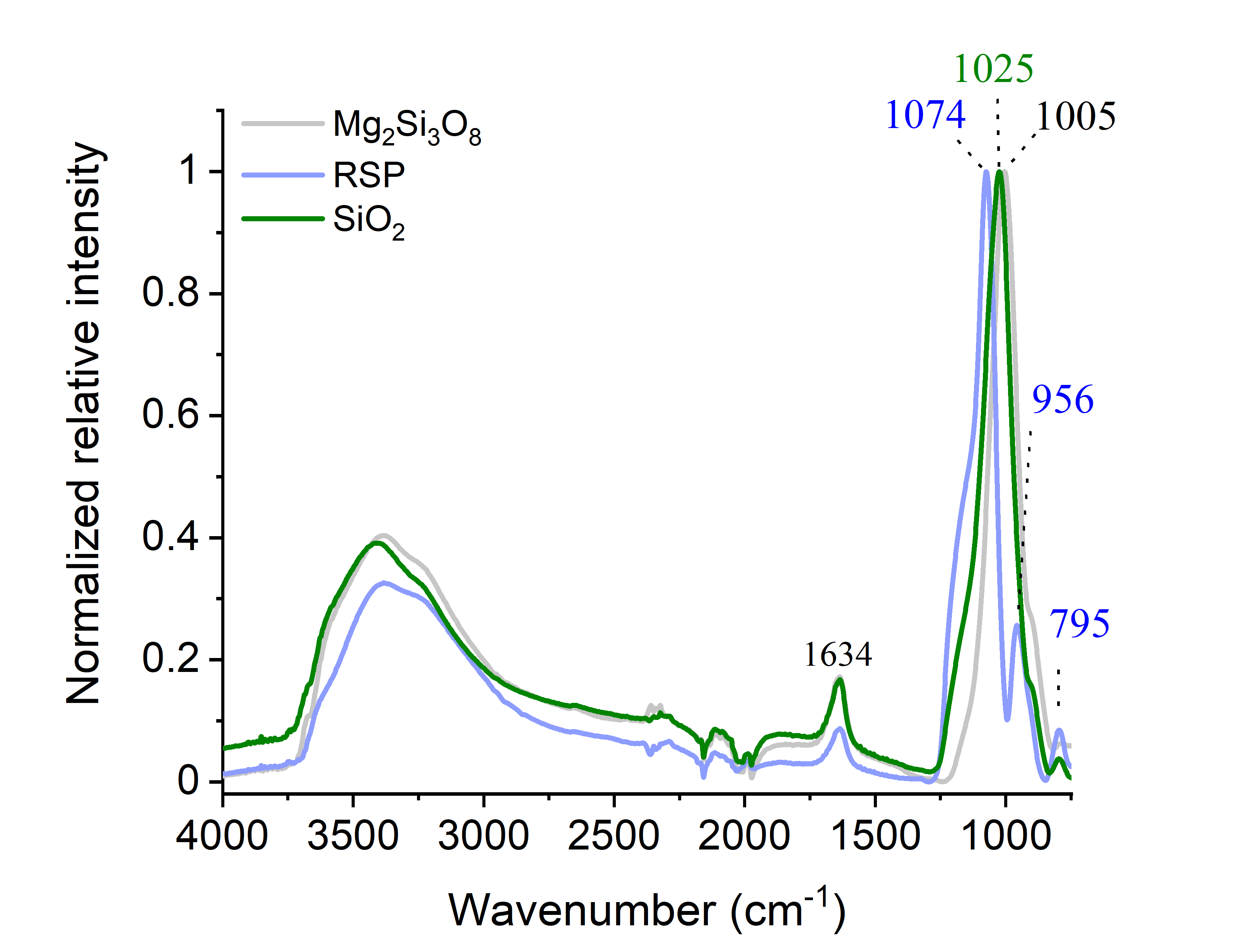


Figure 16. FTIR full spectra of the starting material Mg_2_Si_3_O_8_, (grey trace), the residual silicate phase (RSP) collected after 24 hrs of electrolysis (light blue trace) and amorphous SiO_2_ recovered after pH adjustment (green trace)


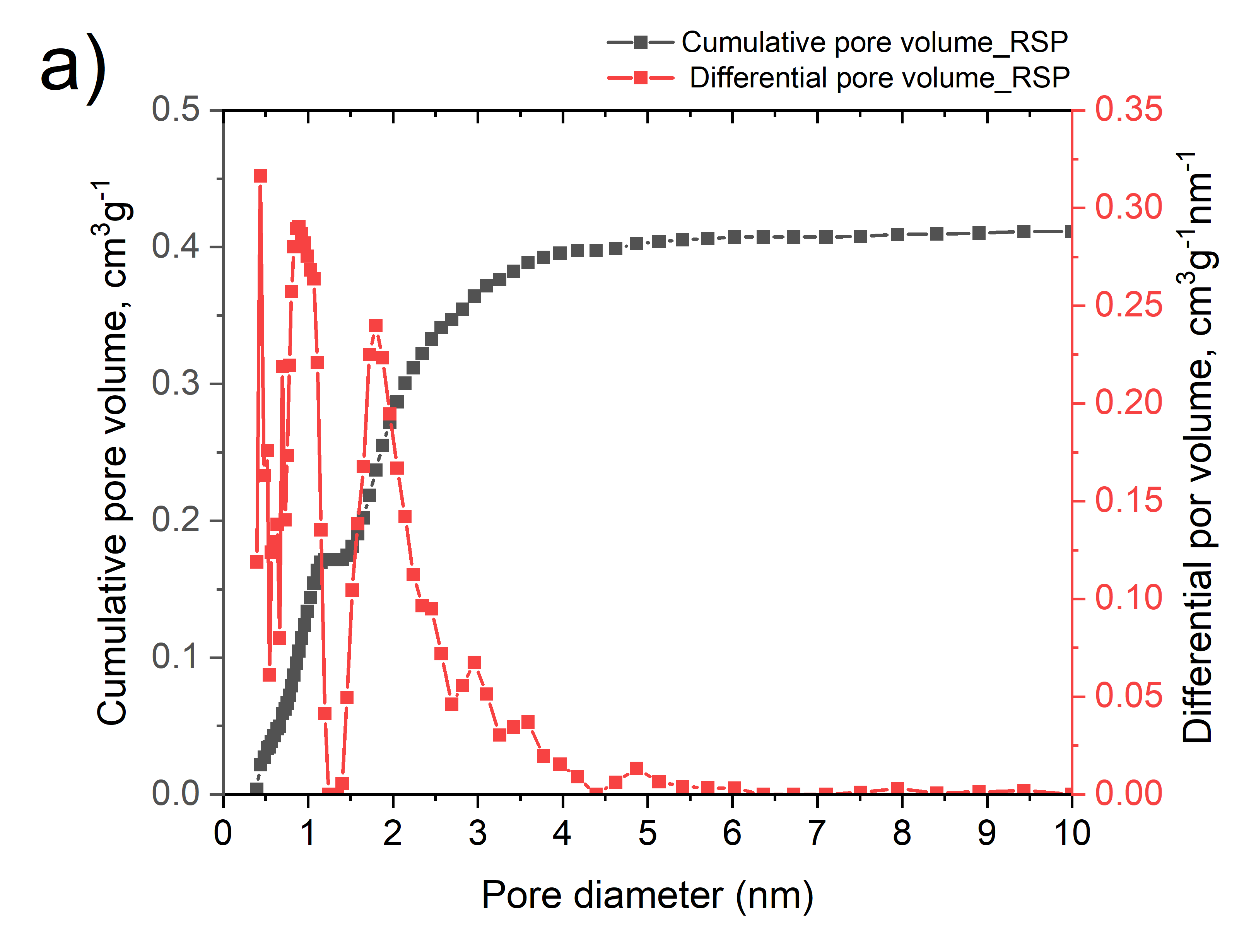

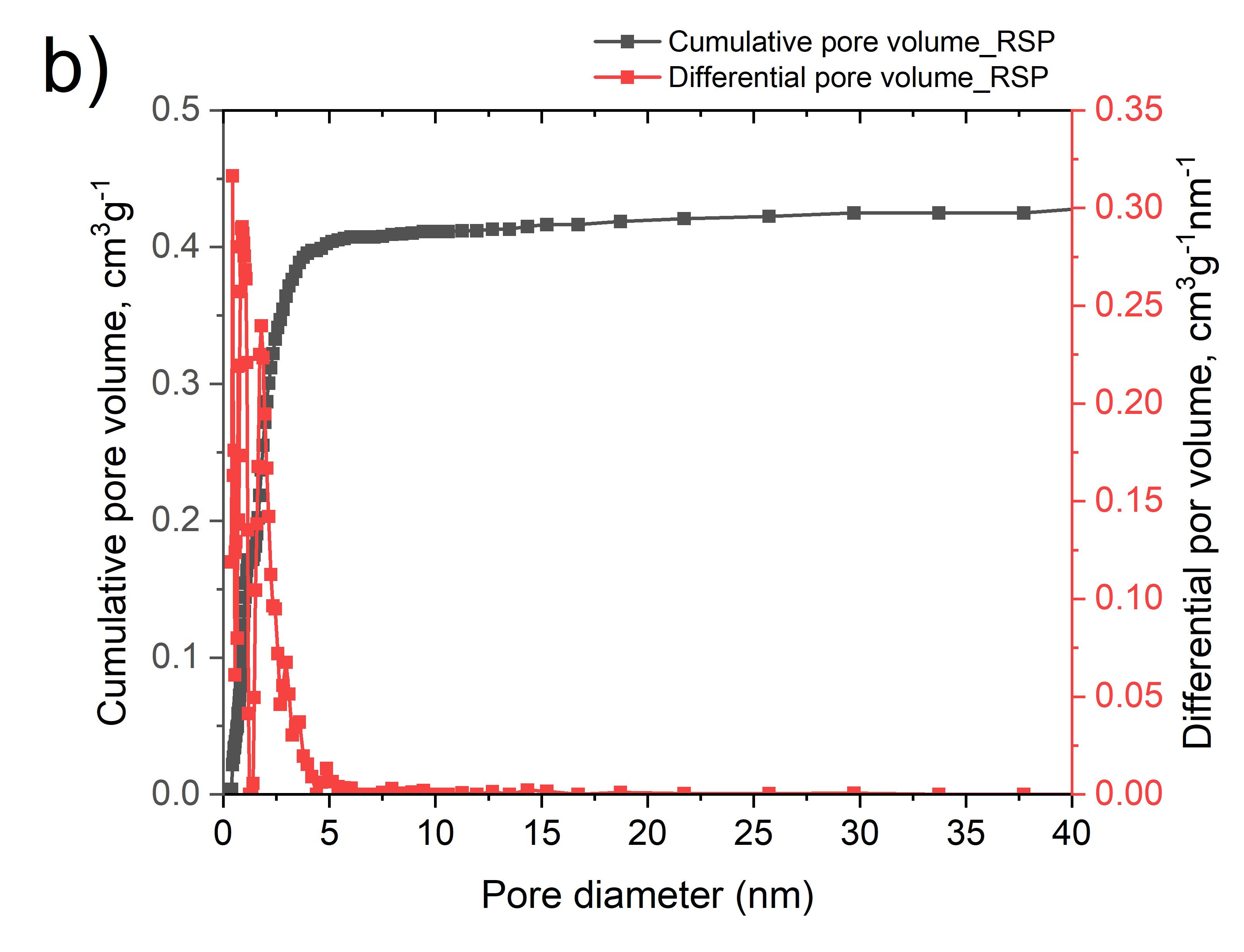


Figure 17. Pore size distribution graphs including cumulative pore volume and differential pore volume for RSP (a) and (b) at different pore diameter scale for clarity. The distributions were calculated from the nitrogen adsorption isotherms using the NLDFT cylindrical pore model for N_2_ 77 K.


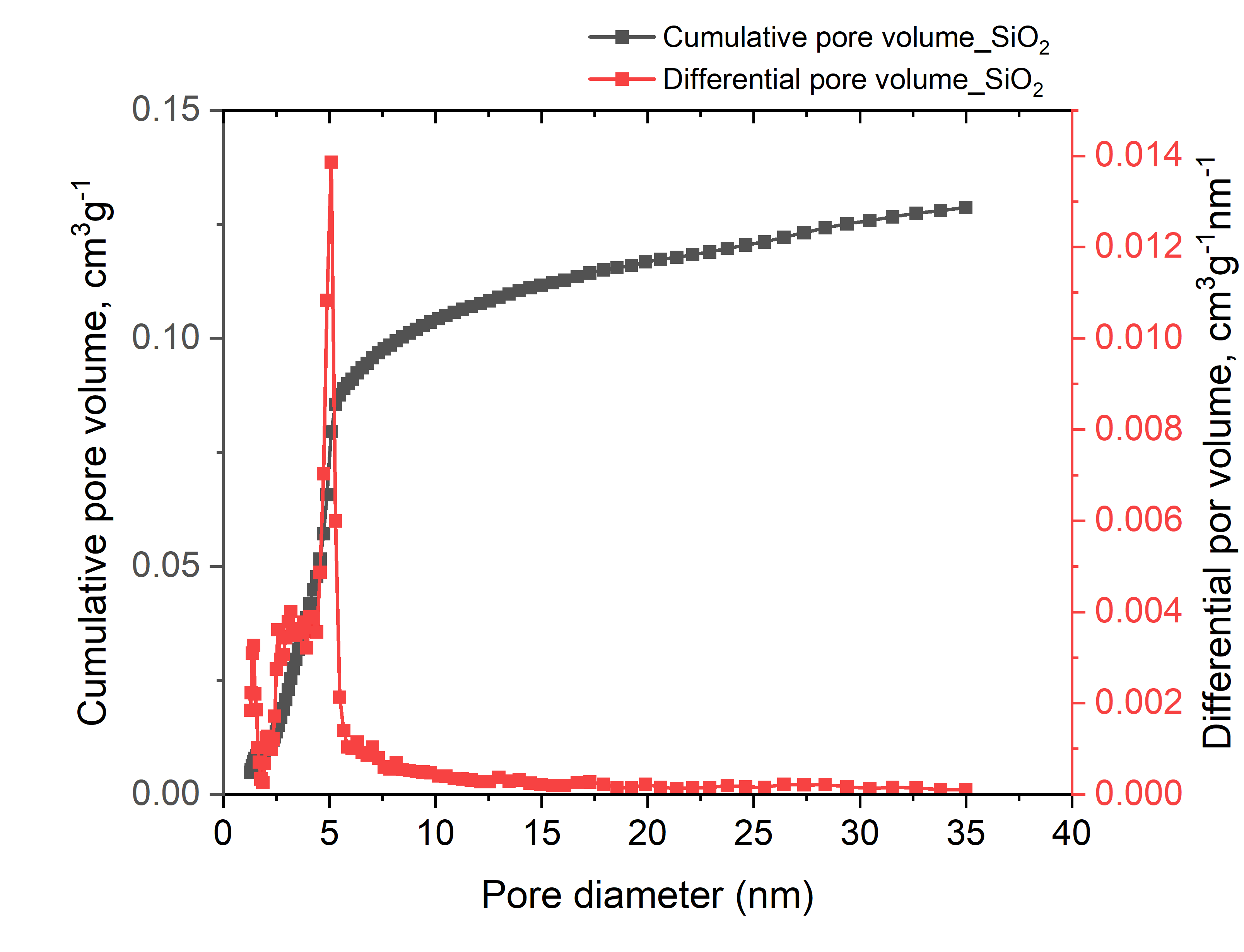


Figure 18. Pore size distribution graphs including cumulative pore volume and differential pore volume for the recovered amorphous SiO_2_. The distributions were calculated from the nitrogen adsorption isotherms using the NLDFT cylindrical pore model for N_2_ 77 K.

# Substrate Characterization


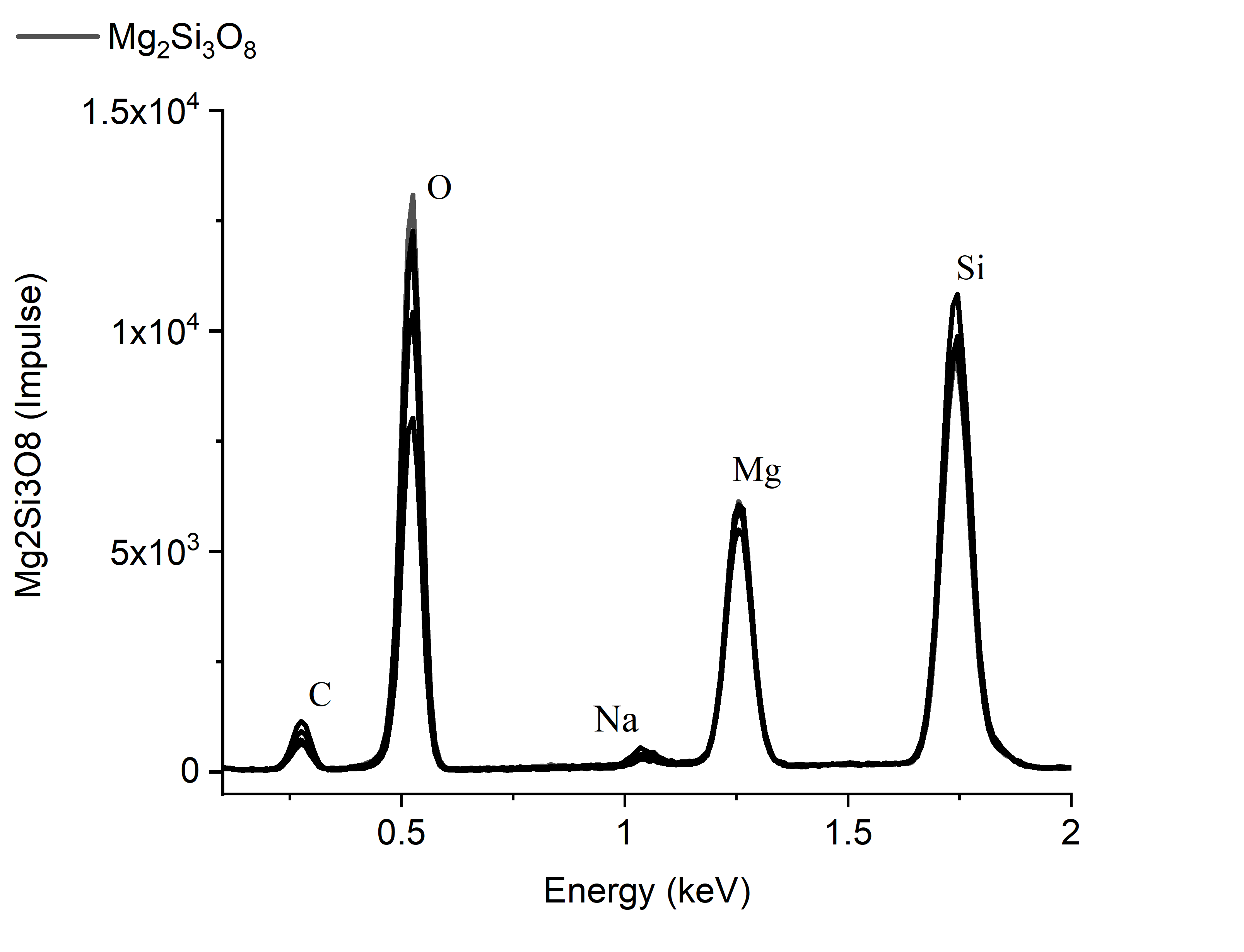


Figure 19. EDX spectrum of the magnesium trisilicate starting material (Mg_2_Si_3_O_8_).


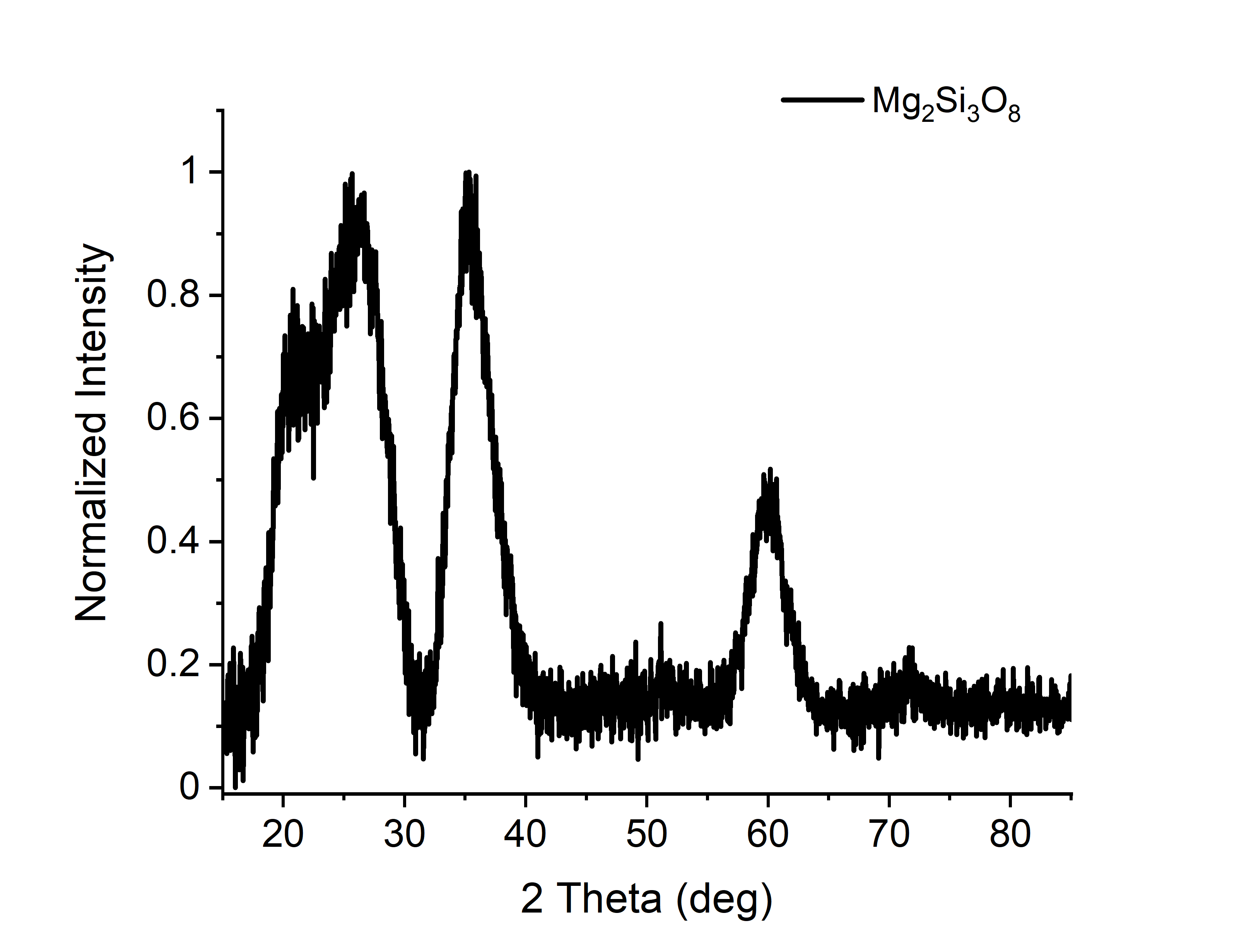


Figure 20. XRD diffraction pattern of the magnesium trisilicate staring material (Mg_2_Si_3_O_8_). The XRD displays broad peaks suggesting a high degree of amorphicity. These peaks at similar 2θ positions compared to poorly crystalline M-S-H phases reported previously. ^6-8^


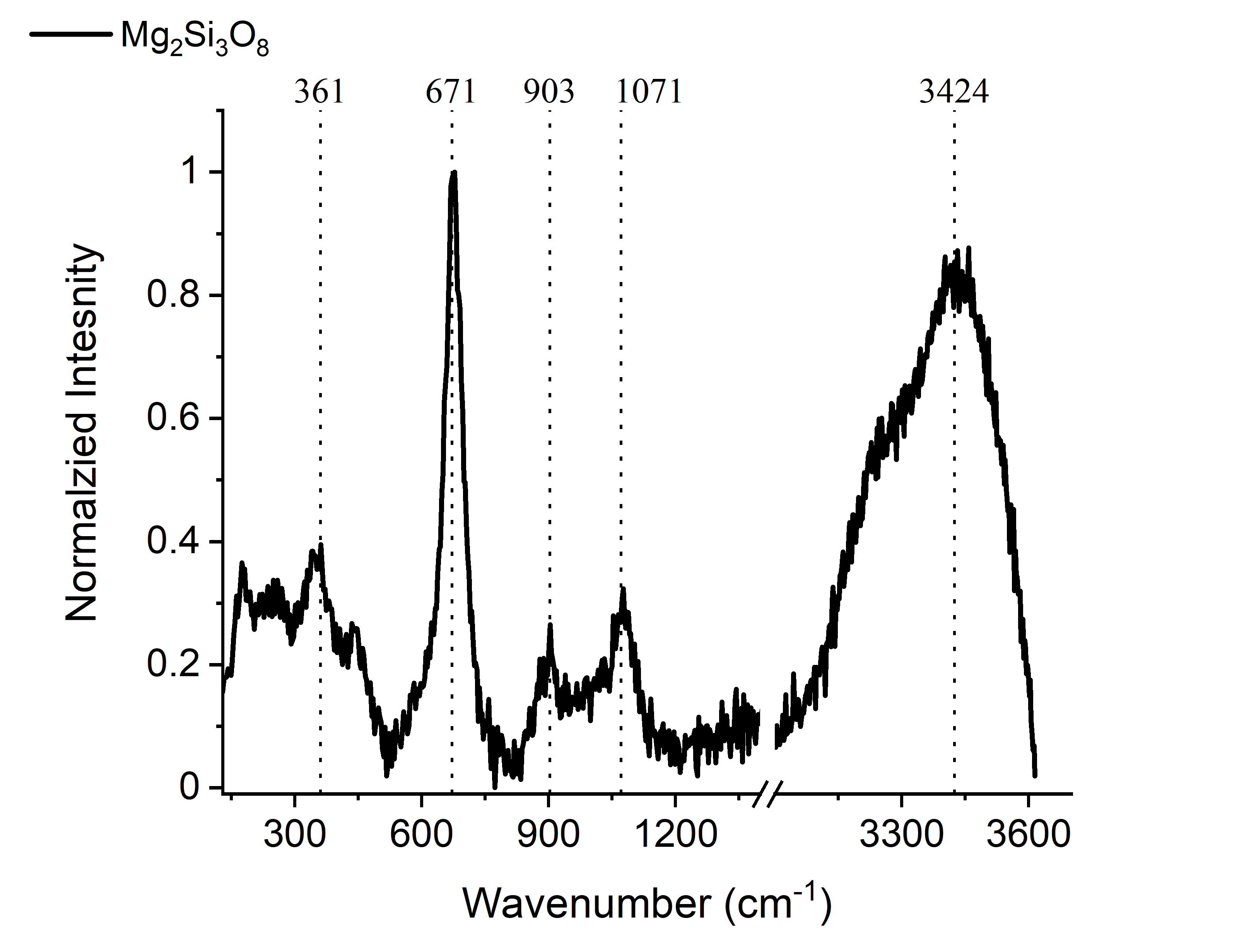


Figure 21. Raman spectrum at 514 nm excitation wavelength of the magnesium trisilicate staring material (Mg_2_Si_3_O_8_). The bands centering at approximately 361 cm^-1^ are attributed to the octahedral O_6_ vibrations of the silicate, the large band centered at 671 cm^-1^ corresponds to the tetrahedral O_4_ ring vibrations of the silicate and the bands at 903 and 1071 cm^-1^ are attributed to the tetrahedral O_4_ stretching vibrations of the silicate.^9^ The broad band at 3424 cm^-1^ is attributed to O—H stretching vibrations, likely from adventitious H_2_O.

# Electrolysis set-up and electrode characterization


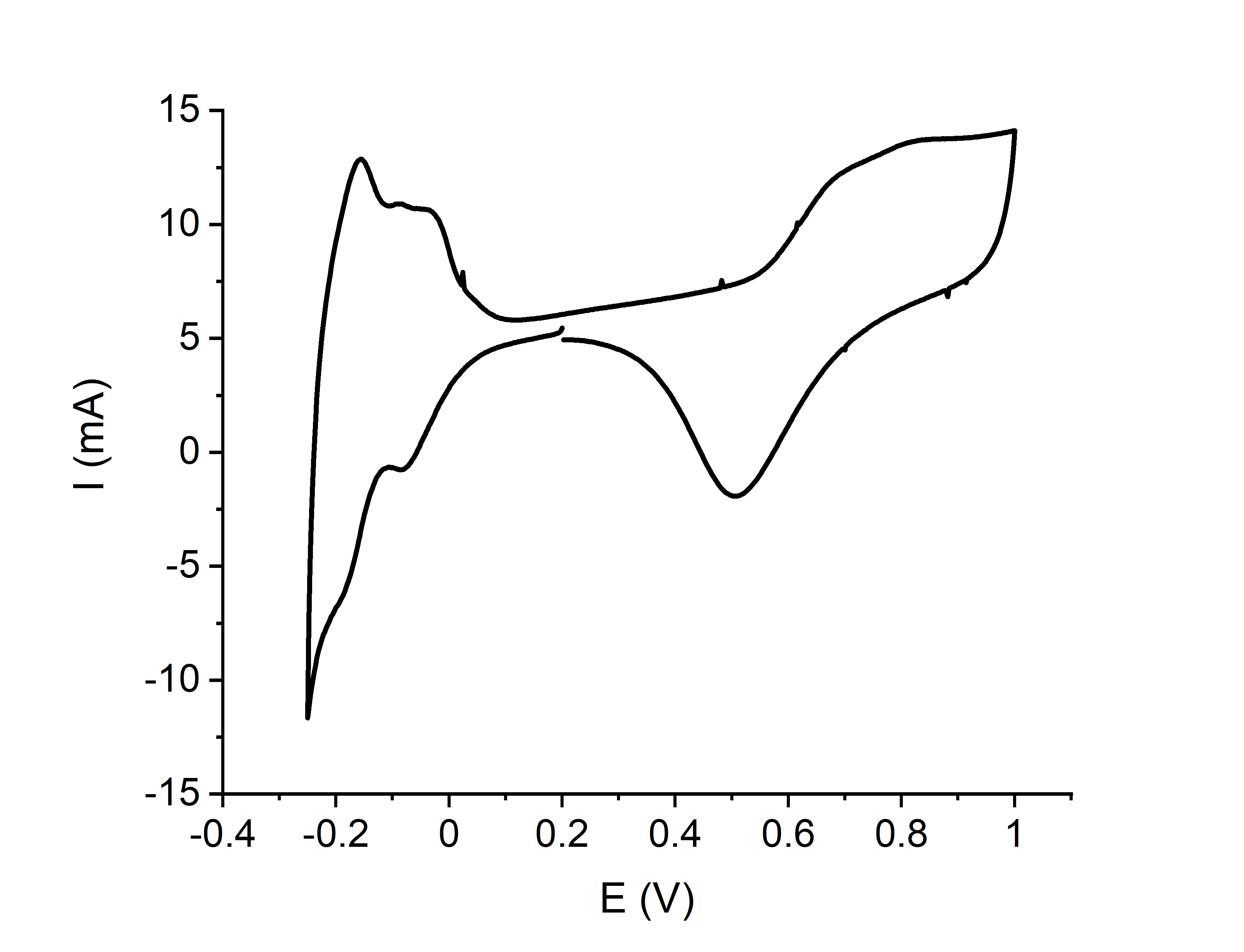

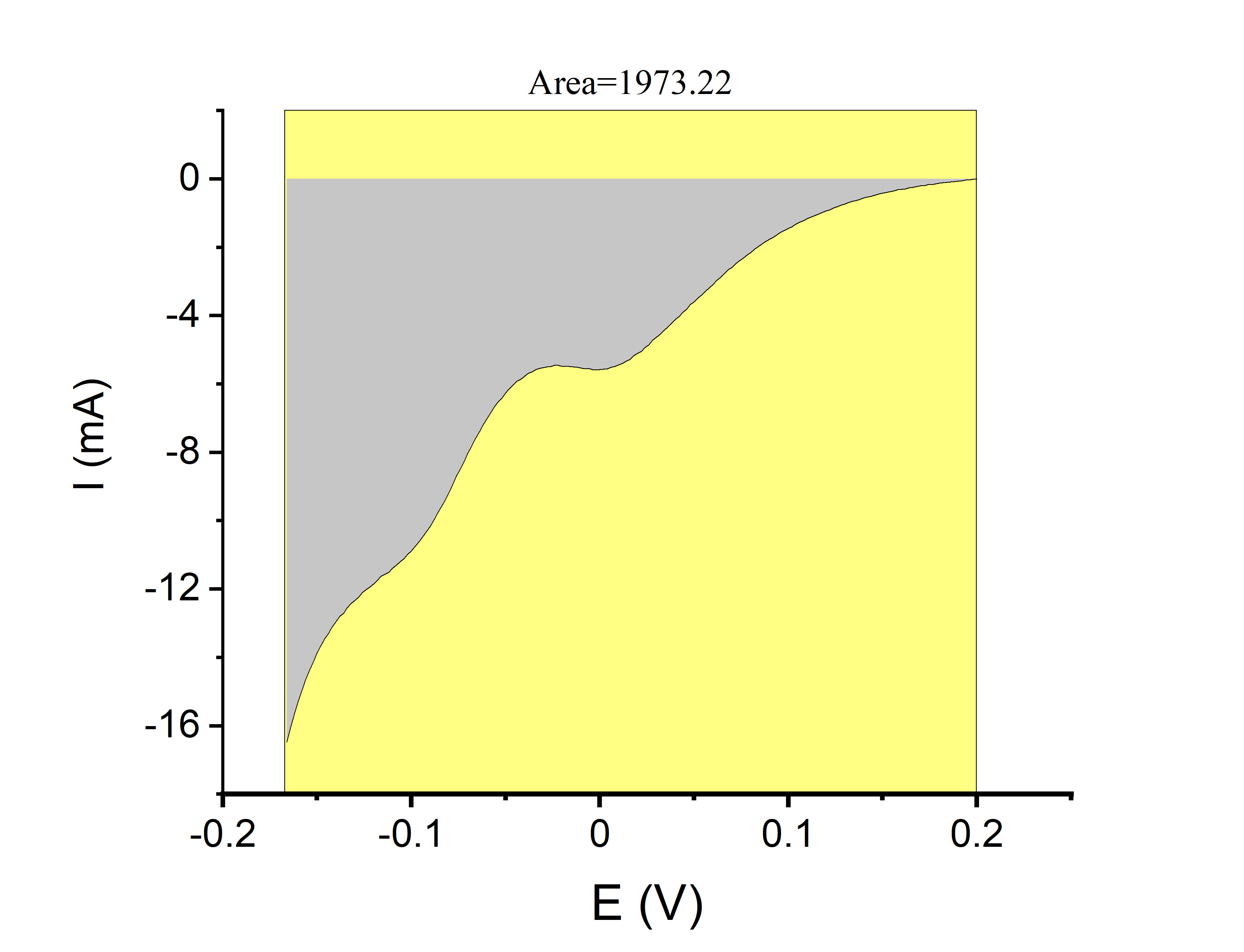


Figure 22. (Left) Cyclic voltammetry trace of Pt mesh electrode in 0.5 M H_2_SO_4_ at 100 mV/s. (Right) integrated area of hydrogen adsorption peak after correcting for capacitive current.


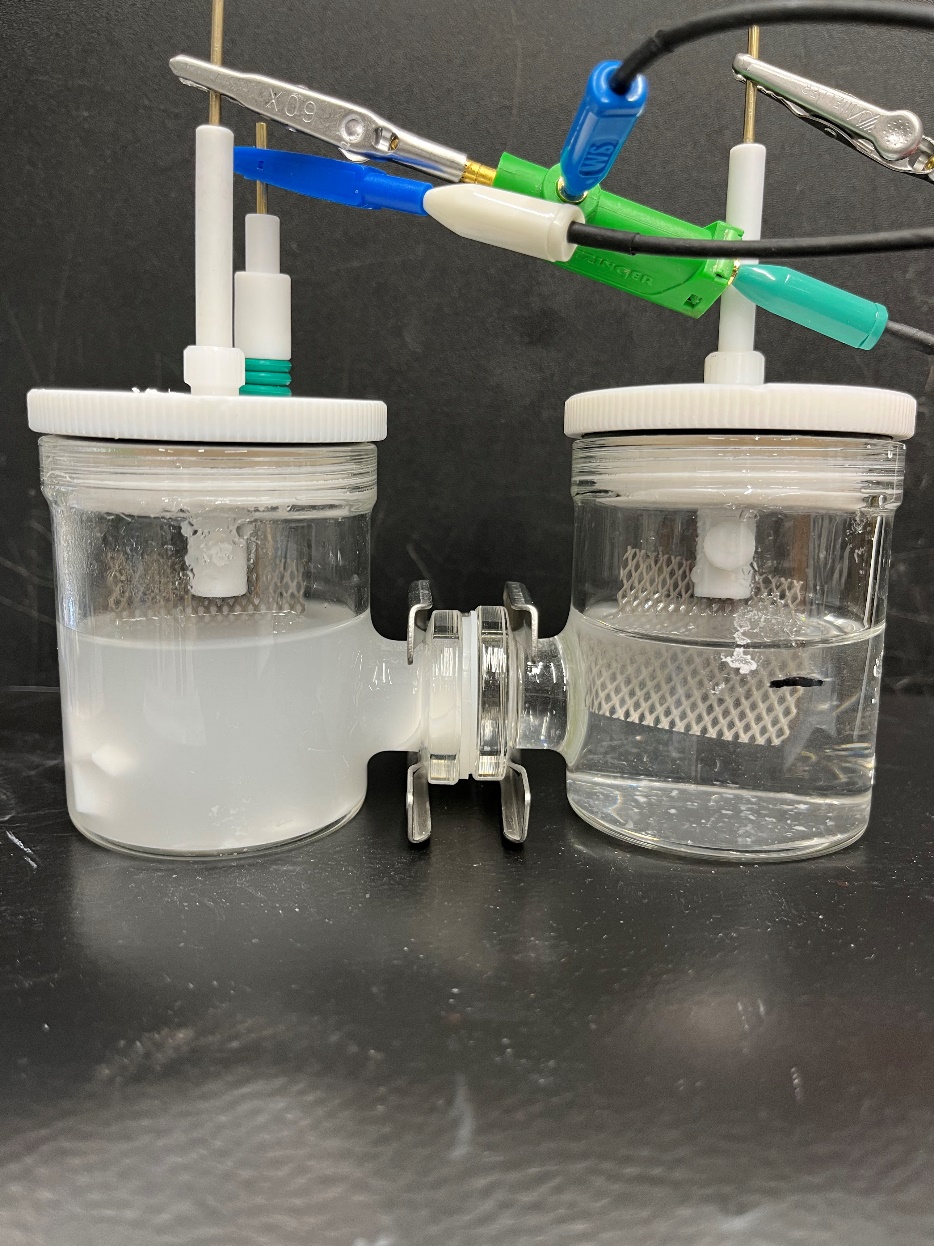


Figure 23. A photo of the electrochemical “H” cell after 4 hrs of electrolysis. The anode is situated in the left compartment along with 1.0 g of starting materials (Mg_2_Si_3_O_8_). The cathode is situated in the right compartment where Mg(OH)_2_ is observed to precipitate. The two compartments are separated by a Nafion 115 membrane.


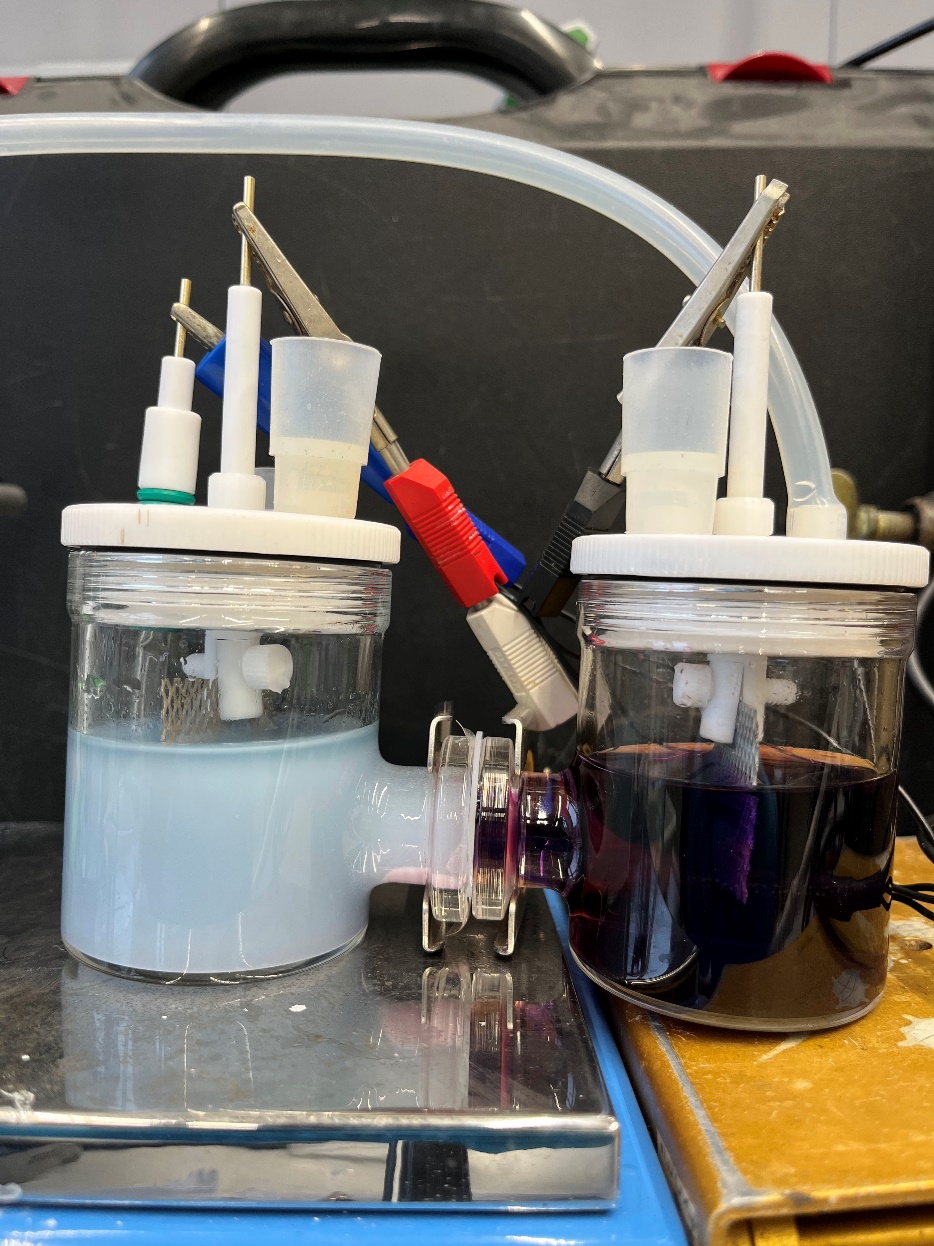


Figure 24. A photo of the electrochemical “H” cell in the presence of a pH indicator at the beginning of electrolysis (5 min). The anode is situated in the left compartment along with 1.0 g of starting materials (Mg_2_Si_3_O_8_). The cathode is situated in the right compartment. The two compartments are separated by a Nafion 115 membrane. The cathodic compartment is violet, indicating a highly basic environment while the anodic compartment is blue, due to the inherent basicity of the Mg_2_Si_3_O_8_.

S


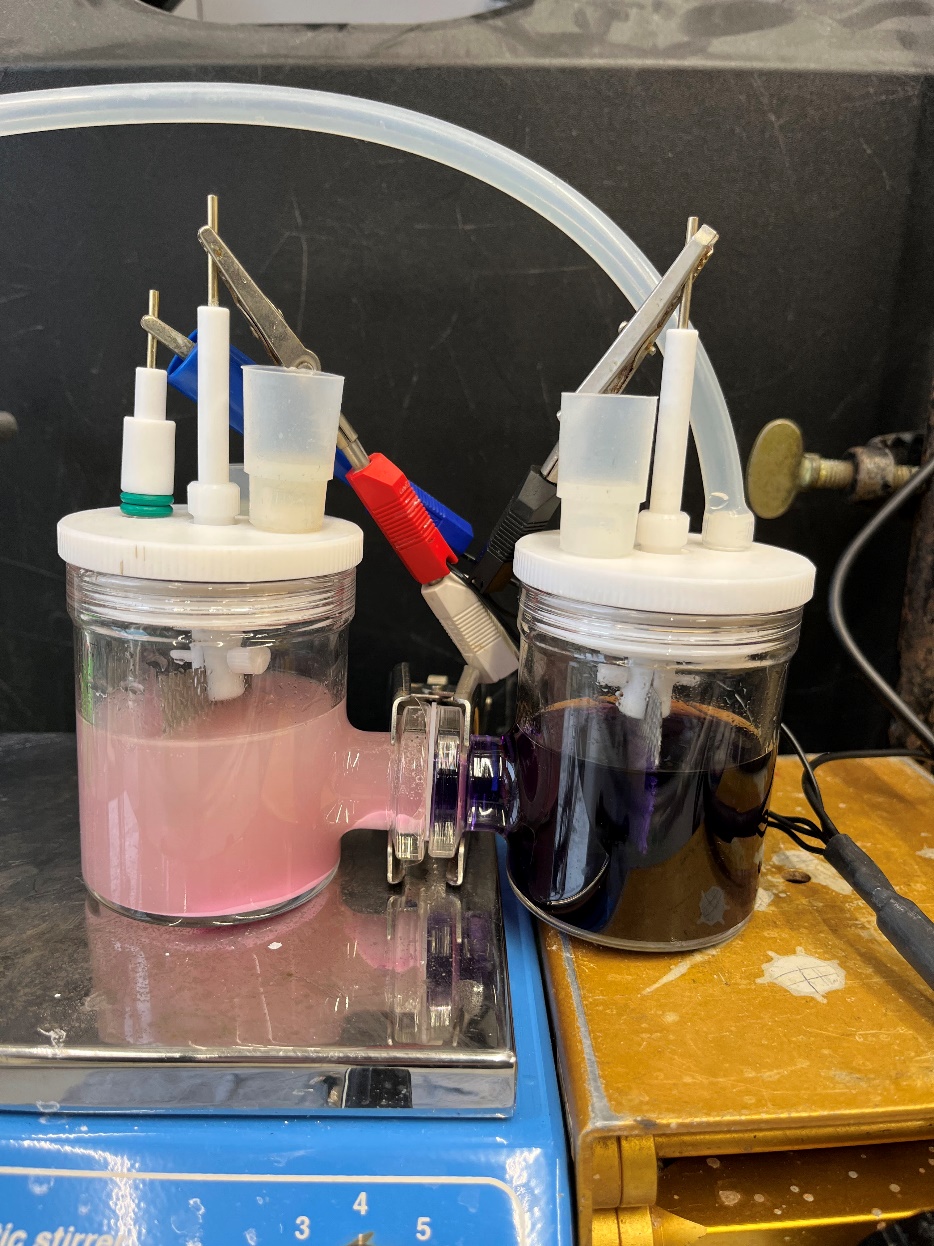


Figure 25. A photo of the electrochemical “H” cell in the presence of a pH indicator after 4 hrs of electrolysis. The anode is situated in the left compartment along with 1.0 g of starting materials (Mg_2_Si_3_O_8_). The cathode is situated in the right compartment. The two compartments are separated by a Nafion 115 membrane. The cathodic compartment is violet, indicating a highly basic environment while the anodic compartment has shifted to a red/pink color as the acidic environment generated at the anode begins to dissolve the Mg_2_Si_3_O_8_.

# References:

1. Buchanan, R. A.; Caspers, H. H.; Murphy, J., Lattice Vibration Spectra of Mg(OH)2 and Ca(OH)2. *Appl. Opt.* **1963,** *2* (11), 1147-1150.

2. Andrews, L.; Wang, X., Infrared Spectra of the Group 2 Metal Dihydroxide Molecules. *Inorganic Chemistry* **2005,** *44* (1), 11-13.

3. Williams, Q.; Collerson, B.; Knittle, E., Vibrational spectra of magnesite (MgCO3) and calcite-III at high pressures. *American Mineralogist* **1992,** *77* (11-12), 1158-1165.

4. Dekermenjian, M.; Ruediger, A. P.; Merlen, A., Raman spectroscopy investigation of magnesium oxide nanoparticles. *RSC Advances* **2023,** *13* (38), 26683-26689.

5. Rutt, H. N.; Nicola, J. H., Raman spectra of carbonates of calcite structure. *Journal of Physics C: Solid State Physics* **1974,** *7* (24), 4522.

6. Walling, S. A.; Provis, J. L., Magnesia-Based Cements: A Journey of 150 Years, and Cements for the Future? *Chem. Rev.* **2016,** *116* (7), 4170-4204.

7. Cornu, D.; Lin, L.; Daou, M. M.; Jaber, M.; Krafft, J.-M.; Herledan, V.; Laugel, G.; Millot, Y.; Lauron-Pernot, H., Influence of acid–base properties of Mg-based catalysts on transesterification: role of magnesium silicate hydrate formation. *Catalysis Science & Technology* **2017,** *7* (8), 1701-1712.

8. Walling, S. A. Conversion of magnesium bearing radioactive wastes into cementitious binders. Unviersity of Sheffield, 2016.

9. Aspiotis, S.; Schlüter, J.; Hildebrandt, F.; Mihailova, B., Raman spectroscopy for crystallochemical analysis of Mg-rich layered silicates: Serpentine and talc. *Journal of Raman Spectroscopy* **2023,** *54* (12), 1502-1516.
